# Genomics Reveal Admixture and Unexpected Patterns of Diversity in a Parapatric Pair of Butterflies

**DOI:** 10.3390/genes12122009

**Published:** 2021-12-17

**Authors:** Mohadeseh Sadat Tahami, Vlad Dincă, Kyung Min Lee, Roger Vila, Mukta Joshi, Maria Heikkilä, Leonardo Dapporto, Sarah Schmid, Peter Huemer, Marko Mutanen

**Affiliations:** 1 Ecology and Genetics Research Unit, University of Oulu, P.O. Box 3000, 90014 Oulu, Finland; vlad.e.dinca@gmail.com (V.D.); kyung.m.lee@helsinki.fi (K.M.L.); muktajoshi3@gmail.com (M.J.); marko.mutanen@oulu.fi (M.M.); 2 Institut de Biologia Evolutiva (CSIC—Universitat Pompeu Fabra), Passeig Marítim de la Barceloneta, 37, 08003 Barcelona, Spain; roger.vila@csic.es; 3 Zoology Unit, Finnish Museum of Natural History, University of Helsinki, P.O. Box 17, 00014 Helsinki, Finland; maria.heikkila@helsinki.fi; 4 Numerical and Experimental Zoology Laboratory (ZEN Lab), Dipartimento di Biologia, Dell’ Università di Firenze, Via Madonna del Piano 6, 50019 Sesto Fiorentino, Italy; leonardo.dapporto@unifi.it; 5 Departement de Biologie Computationnelle, Faculte de Biologie et Medecine, Universite de Lausanne, 1015 Lausanne, Switzerland; sarah.schmid@unil.ch; 6 Tiroler Landesmuseen Betriebsges.m.b.H., Naturwissenschaftliche Sammlungen, Krajnc-Str. 1, A-6060 Hall, Austria; p.huemer@tiroler-landesmuseen.at

**Keywords:** ddRAD sequencing, parapatry, paraphyletic species, genomic admixture, *Melitaea*, species delimitation

## Abstract

We studied the evolutionary relationship of two widely distributed parapatric butterfly species, *Melitaea athalia* and *Melitaea celadussa*, using the ddRAD sequencing approach, as well as genital morphology and mtDNA data. *M. athalia* was retrieved as paraphyletic with respect to *M. celadussa*. Several cases of mito-nuclear discordance and morpho-genetic mismatch were found in the contact zone. A strongly diverged and marginally sympatric clade of *M. athalia* from the Balkans was revealed. An in-depth analysis of genomic structure detected high levels of admixture between *M. athalia* and *M. celadussa* at the contact zone, though not reaching the Balkan clade. The demographic modelling of populations supported the intermediate genetic make-up of European *M. athalia* populations with regards to *M. celadussa* and the Balkan clade. However, the dissimilarity matrix of genotype data (PCoA) suggested the Balkan lineage having a genetic component that is unrelated to the *athalia*-*celadussa* group. Although narrowly sympatric, almost no signs of gene flow were found between the main *M. athalia* group and the Balkan clade. We propose two possible scenarios on the historical evolution of our model taxa and the role of the last glacial maximum in shaping their current distribution. Finally, we discuss the complexities regarding the taxonomic delimitation of parapatric taxa.

## 1. Introduction

Difficulties to delimit species under many circumstances are severely undermining attempts to catalogue global biodiversity and have an adverse effect on conservation efforts [1]. These difficulties stem from both biological and operational causes. As speciation is not an event but a process, defining the time of birth of a new species remains inherently arbitrary [2]. Ontological difficulties, in turn, are largely due to the lack of consensus over the species concept and critical properties that a population should bear in order to be considered as a valid species [3]. Additionally, the critical properties of species can emerge at different time and order during the course of divergence [4]. Although attempts have been made, a widely accepted consensus over the criteria of species is evidently hard to reach. Traditionally, insect species have largely been circumscribed based on morphological traits, assuming that morphological differences, particularly those in genitalia, play a key role in preventing gene flow between populations undergoing speciation [5]. The “lock-and-key hypothesis”, i.e., an idea that genital differences between species have evolved in order to prevent insemination across species [6], was long popular particularly among entomologists, although it suffers from both theoretical weaknesses [7,8] and a lack of empirical support [2,9,10].

The parapatric model of distribution, in which ranges of closely related taxa confront but show only narrow areas of overlap, presents particular challenges for species delimitation. The parapatric distribution pattern may result from parapatric speciation itself, but also from secondary contact of formerly separated and genetically diverged populations [2]. Parapatric taxa represent an appealing condition to study gene flow among emerging species as it is likely a frequently occurring phase in the course of speciation. To obtain a better perspective of relationships of parapatric species, the spatial component and historical dimension of isolation should be taken into account [11]. Indeed, parapatry could have been achieved very recently, notably in the last interglacial, between taxa that remained in allopatry during most of their evolutionary history, with no or only casual contacts.

Populations with a contact zone can be used as a natural experiment to investigate features that hinder or fully prevent gene flow between them. Parapatry is not uncommon, among the best documented, being that of the Hooded Crow (*Corvus cornix*) and the Carrion Crow (*Corvus corone*), species which show broad genetic admixture, but also strong assortative mating [12]. As the evolutionary trajectory of natural populations is traceable in populations’ gene pool, an in-depth genomic study enables obtaining accurate information about the genetic structure and the demographic history of those populations. Emergent genomics tools also provide high potential for better-standardized and quantifiable criteria to species delimitation under para- and allopatry [13,14].

We aimed to provide genomic insight on the evolutionary dynamics of parapatric populations of the butterflies *M. athalia* (Rottemburg, 1775) and *M. celadussa* Fruhstorfer, 1910 (Nymphalidae). These taxa represent one of the most remarkable cases of parapatry among European butterflies. They are widely distributed in most parts of Europe and western parts of Asia and exhibit a narrow contact zone in France, Switzerland, Italy and Austria, with *M. celadussa* occurring in south and west, and *M. athalia* in north and east of the contact zone [15,16,17,18]. Distinguishing between these two taxa is difficult as they are extremely similar in their external morphology, being only diagnosable by a set of characters in their male genitalia. However, specimens with intermediate male genital characters have been documented in the contact zone, for which reason *M. celadussa* was long considered as a subspecies of *M. athalia* [15,19]. *M. celadussa* was recently proposed as a separate species based on molecular evidence [20,21] and it is currently regarded as such in the latest checklist of European butterflies [22]. However, molecular data for this taxon are based on either a single specimen [20] or restricted to DNA barcodes [18]. We used a high-throughput genomics approach to address the genetic structure and evolutionary history of this parapatric pair of butterflies.

## 2. Materials and Methods

### 2.1. Sample Data

The sampling is representative for the distribution of *M. athalia* and *M. celadussa* in Europe, including their contact zone. DNA extraction was performed for 61 specimens of *M. athalia* and *M. celadussa*, as well as for 10 specimens representing outgroup species; *M. caucasogenita/athalia* (see comment in the Supplementary Material), *M. britomartis* Assmann, 1847, *M. deione* (Geyer, [1832]), *M. aurelia* Nickerl, 1850. We assigned individuals to putative species based on established differences in DNA barcodes (the 5′ region of mitochondrial cytochrome c oxidase subunit 1—COI) since it was the only method of identification that was available for all specimens. Additionally, whenever possible, we used genital structure for species attribution (i.e., males with intact genitalia). For a detailed description of morphological, and mitochondrial data preparation and analyses, please refer to the supplementary material (SM). Additionally, data of 418 specimens of *M. athalia* and *M. celadussa* collected across their European distribution and sequenced for DNA barcodes were used to complement and optimize our study (Table S5). The COI tree (Figure S1) and details of voucher specimens (Table S1) are available in SM.

### 2.2. Genomic Assembly and Bioinformatics

After the library preparation and sequencing (please refer to the supplementary material for details), raw demultiplexed Illumina (San Diego, CA, USA) paired-end reads were merged using PEAR [23] with default parameter values. Merging paired-end reads to single-end minimizes false-positive results and generates highly accurate single-end reads. To rule out the potential effect of bacterial contamination and maternally inherited mitochondrial genes, merged reads that were mapped to *Wolbachia pipientis* (GenBank: NZ_JQAM01000001) and *Melitaea cinxia* mitochondrial genome (GenBank: CM002851) were removed using Geneious Prime 2019.2.1 (https://www.geneious.com (accessed on 16 September 2019)). Merged reads were processed as input data for the ipyrad pipeline version 0.9.31 [24].

Cleaned reads were loaded and filtered from adapters and primers through step 1 and 2 of ipyrad. To explore the ddRAD data, several assembly branches were created to test for various levels of consensus quality by applying parameters change. Clustering threshold (c = 80, 85, 88, 90, 93, 94, 95) and minimum number of samples per locus (m = 4, 8, 10, 12, 20) were tested independently and as combinations with each other using both de novo and reference assembly methods. The latter method assembles loci by mapping the paired reads onto the *M. cinxia* (Linnaeus, 1758) complete genome (GCA_00071638). After clustering and aligning reads (step 3), consensus allele sequences were estimated (steps 4 and 5). Samples with consensus coverage lower than 5% were excluded from downstream analyses. Subsequently, other parameters that can affect the quality of the final consensus alignment were also tested as follows: maximum number of SNPs per locus (max_SNPs_locus = 12, 15, 20, 22, 25), maximum number of indels per final locus (max_Indels_locus = 4, 6, 8), maximum fraction of uncalled bases in the consensus sequences (max_Ns_consens = 3, 5, 7), maximum fraction of heterozygous bases allowed in the consensus sequences (max_Hs_consens = 6, 8, 10), and maximum number of low-quality base calls in a read (max_low_qual_bases = 3–5). The latter parameter was kept below the recommended value of 5 as higher values will increase the final number of ambiguous (N) sites which will affect the accuracy and reliability of downstream clustering. Before clustering the concatenated consensus sequences, one allele was randomly called per locus so that ambiguous characters have a lesser effect on clustering, but the resulting data retained information for heterozygotes (step 6) [24]. The clustered sequences were then aligned and filtered according to the given “m”, “max_SNPs_locus” and other parameters and saved in multiple output formats (step 7).

### 2.3. Phylogenetic Analysis

To study the phylogenetic relationships among taxa, we conducted a maximum likelihood (ML) analysis. We first searched for the best fitting model of sequence evolution using ModelFinder implemented in IQ-TREE version 1.5.4 [25]. The result recovered TVM + F + I + G4 as the best fitting substitution model for our data. The phylogenetic tree was constructed with an ultrafast bootstrap approximation model (1000 replicates) using the same IQ-TREE version [26,27]. The consensus tree was visualized in FigTree v1.4.4 (https://github.com/rambaut/figtree/releases (accessed on 25 November 2019)) and was rooted on *M. aurelia*.

### 2.4. Analyses of Genomic Diversity and Admixture

To understand the genomic patterns and the level of admixture in parapatry, we performed STRUCTURE [28] analysis using Bayesian algorithm. The admixture model was used on SNP data shared between a minimum number of 20 samples (*m*20 and 18,383 generated SNPs) with no prior population assignment. To determine the optimal number of genetic clusters (K), we used the ∆K method in STRUCTURE HARVESTER [29,30] with 500,000 iterations for MCMC and 100,000 as pre-burn in. We tested five putative numbers of clusters, K = 1−5, with 10 iterations for each K out of which the optimum of K = 2 was estimated. We then aligned the cluster assignments of 2–4 across replicate analyses in CLUMPP [31] and used DISTRUCT [32] for visual representations of the aligned clusters. To have a better understanding of the geographic patterns of genomic admixture at K = 2 and K = 3, we mapped the membership coefficient matrix obtained from the Bayesian clustering model to the matrix of geographical coordinates in Jupyter lab (https://jupyter.org (accessed on 23 April 2020)) using the Basemap Matplotlib Toolkit v1.2.0 implemented in the Matplotlib package v3.0.3 [33].

To further investigate levels of divergence within and between clusters, we calculated pairwise F_st_ and F_is_ in R 3.6.1 [34] using the function “basic.stat” of package Hierfstat [35].

### 2.5. Population History

The history of populations was investigated using approximate Bayesian computations in DIYABC v2.1.0 [36] based on SNP data (*m*20). Four historical scenarios were assumed as follows: (1) main *M. athalia* are diverged from the Balkan lineage (see phylogenetic result), (2) both main *M. athalia* and Balkan lineage are diverged from *M. celadussa*, (3) main *M. athalia* is an admixture of the Balkan lineage and *M. celadussa*, and (4) main *M. athalia*, the Balkan lineage and *M. celadussa* split at the same time considering the Balkan lineage as the ancestral population. All scenarios were assumed uniform with priors for population effective size (Ne) and times (t) between 10–10,000, t2 was considered bigger than t1, and the admixture rate (r) between 0.001–0.999. The analysis was performed with 1,000,000 simulations on a subset of 2000 loci out of 10,724.

### 2.6. Isolation by Distance and Geographic Representation of Genetic Differentiation

As genomic data sometimes tend to be clustered geographically, we also tested for the genomic isolation by geographical distance (IBD) based on individual level data. Mantel correlation between genomic and geographic distance was assessed with the “mantel” function of R package adegenet [37] with 999 permutations. Genomic Edwards’ distance matrices were generated using the “dist” function of R. A geographical distance matrix encompassing the minimum path distance among specimens over land was produced by calculating the length of the minimum over-ground path among sequenced specimens using the ”costDistance” function as implemented in the gdistance R package (v. 1.2-1) [38]. The local density was measured using a 2-dimensional kernel density estimation within the R package adegenet. As done with COI (see Supplementary Materials for details), we applied PCoA to the ddRADseq dissimilarity matrix using the “cmdscale” R function and minimized the location and rotation differences with respect to the COI PCoA, then we projected it in the RGB colour space the specimens and mapped the resulting colours.

## 3. Results

### 3.1. Patterns of COI Variability

The neighbor joining (NJ) tree based on DNA barcodes (Figure S1) grouped specimens of *M. athalia*–*M. celadussa* into three main well-supported (Bootstrap Support (BS) > 0.95) clusters. Based on their established distribution [22], one cluster was assigned to *M. celadussa*, and the other two to *M. athalia*. One of the *M. athalia* clusters consisted of six specimens originating from the Balkans and from here on we refer to it as “the Balkan lineage”, and the rest of *M*. *athalia* will be referred to as “main *M. athalia*”. The minimum p-distances between the three main clusters were: main *M. athalia*–*M. celadussa* = 2.75%; Balkan lineage–main *M. athalia*: 3.92%; Balkan lineage–*M. celadussa*: 3.92%. It should be noted that the main *M. athalia* cluster also included the specimen that we tentatively identified as *M. caucasogenita/athalia* (see comment in the supplementary material) (Figure S1). 

### 3.2. Morphometrics

**Figure 1:**
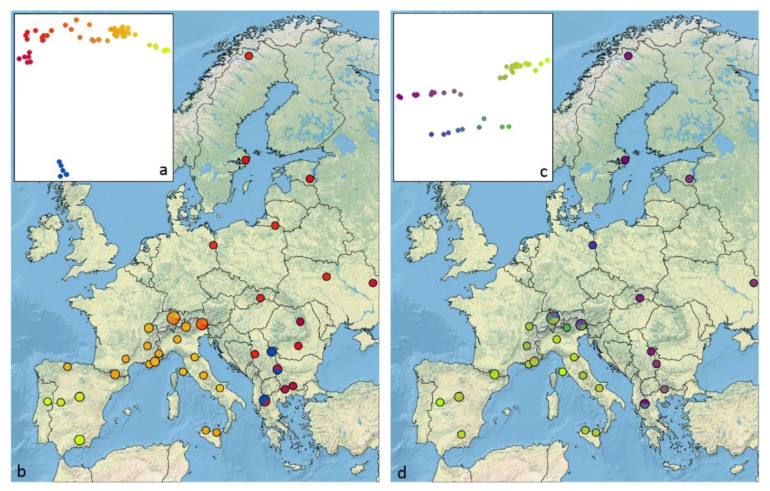
Patterns of genetic and morphological differentiation in *M. athalia* and *M. celadussa*: (**a**) The configuration obtained after PCoA of Edwards’ distances calculated on ddRAD data and projected in RGB space where similar individuals are characterised by similar colours; (**b**) The geographic location of each specimen with the colour obtained in the PCoA; (**c**) The configuration obtained after PCA on genitalia traits; (**d**) The geographic location of specimens.

The measured elements of the male genitalia (sub-unci and posterior process of the valva) (Figure S2) revealed a morphological continuum with an overlap between *M. celadussa* and *M. athalia* (Figure 1c and Figure S3, Table S2), while the degree of bifurcation of the posterior process of valva showed a higher concordance with the DNA barcodes. The length of the posterior process of valva showed considerable overlap between taxa, although specimens attributable to *M. celadussa*, based on DNA barcodes, tended to have a longer posterior process (Table S2, Figure S3a). *M. celadussa* also had absent or vestigial sub-unci, as opposed to the longer sub-unci in main *M. athalia* and the Balkan lineage (Figure S3). However, this pattern was broken by six specimens with intermediate length of sub-unci (north-eastern Spain, south-eastern France and Switzerland), as well as by six specimens (Switzerland and northern Italy) which had a genitalia morphology of *M. athalia* type, but belonged to *M. celadussa* (Table S2, Figure S3b). The three measured specimens belonging to the Balkan COI lineage had genitalia morphology of *M. athalia* type (i.e., well-developed sub-unci and shorter posterior process of the valva) (Figure S3).

The presence or absence of a bifurcation on the posterior process of the valva (Table S2, Figure S3a) was better correlated with COI identification than the length of the sub-unci. Indeed, all specimens with COI of *M. celadussa* showing intermediate sub-unci had no bifurcation except for one specimen showing a small bifurcation. All specimens with genitalia of *M. athalia* type had a bifurcation, including all those attributable to *M. celadussa* based on COI and those belonging to the Balkan COI lineage. Accordingly, a PCA identified two components with eigenvalue higher than one (Figure S4) where longer sub-unci were highly correlated with the presence of a larger bifurcation. The projection of this configuration on RGB colour and the location of obtained colours on the map revealed that the individuals with intermediate genitalic morphology and/or incongruent mito-morphology are only seen in the contact zone of the Alps (Figure 1d).

### 3.3. Overview of ddRAD Data

An average of 1.8 million raw reads were obtained from Illumina sequencing (Table S3). After checking results from different combinations of parameter values for clustering using both de novo and reference assembly methods, we chose 85% (*c*0.85) of sequence similarity to retain loci shared across more than four samples (*m*4) for phylogenetic analysis along with the default values for other parameters. This approach recovered a robust sequence alignment with confident summary statistics. The tree topologies reconstructed from either of the assembly methods with the selected parameter values were generally congruent. Although the de novo assembly could recover a higher number of loci and SNPs, we chose the reference-based assembly as it yielded a more robust tree. Generally, it has been recommended to map restriction site associated genomic data to a reference genome to avoid potential collecting of repeated regions and non-informative loci [39]. We retrieved a total of 3,892,196 base pairs (bp) with 30,696 (0.8%) identical sites, 37,507 putative orthologous loci and 204,430 SNPs of which 71 774 are parsimony informative. The minimum percentage of sample coverage was 6.23% and the maximum was 24.13%, while the proportion of missing data was 85.9% over all loci. For genomic analyses other than phylogeny, we used the *c*0.85-*m*20 data assembly, therefore significantly lowering the proportion of missing data to below 50% (41%) and increasing the average sample coverage to 77.81%.

### 3.4. ddRAD Phylogeny

**Figure 2:**
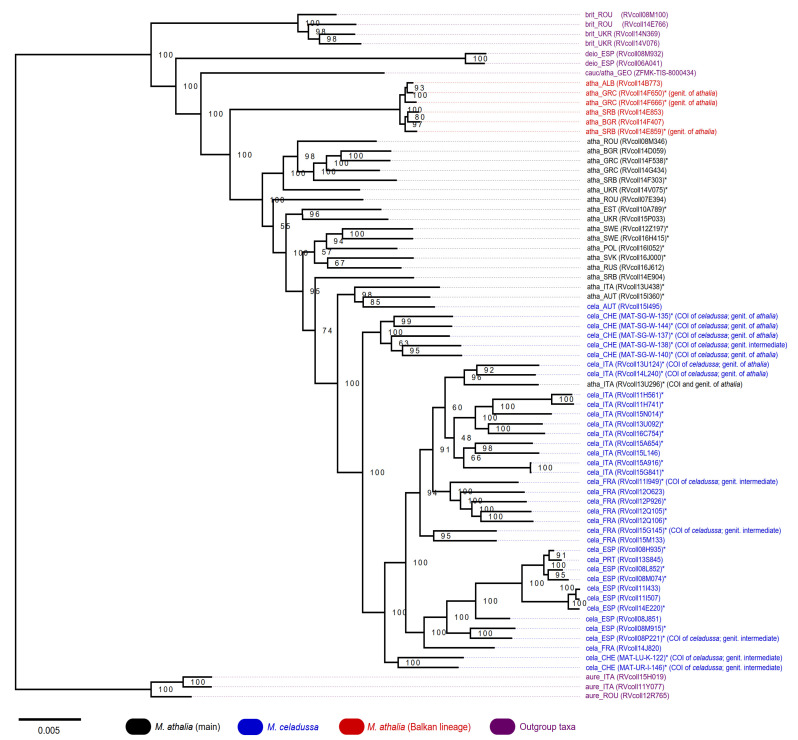
Maximum likelihood tree of *M. athalia*−*M. celadussa* inferred based on ddRAD data using reference assembly. Bootstrap supports are given at each node. The tree is rooted on *M. aurelia*. Asterisks indicate specimens examined for morphometrics.

The ML tree reconstructed based on concatenated ddRAD loci revealed a grouping of specimens that is discordant to the taxonomic assignments based on COI and morphology (Figure 2). In a tree rooted to *M. aurelia*, the putative specimen of *M. caucasogenita/athalia* was found as a sister lineage to the well supported (BS = 100) *M. athalia*/*celadussa* clade. Specimens of *M. athalia* formed a highly paraphyletic grade with respect to *M. celadussa*. Remarkably, a lineage consisting of six Balkan specimens attributed to *M. athalia* was recovered as a sister lineage to all other specimens of *M. athalia* and *M. celadussa* (BS = 100). *M. celadussa* formed a near-monophyletic clade, albeit with the following two exceptions. The specimen RVcoll13U296 recognized as *M. athalia* by both genitalia morphology and COI was nested within this clade. Similarly, one specimen of *M. celadussa* COI (RVcoll15I495) was recovered among two specimens of *M. athalia* (RVcoll13U438 and RVcoll15I360). Within the clade of *M. celadussa*, five Swiss specimens, each showing mito-morphological discordance (Figures S1 and S3, Table S2), formed a relatively divergent sister clade to the rest of *M. celadussa* (plus RVcoll13U296). This Swiss clade did not show a clear separation from most specimens of *M. celadussa* in the COI tree (Figure S1). Other cases of intermediate genitalia were placed within *M. celadussa*.

### 3.5. Genomic Diversity and Admixture

**Figure 3:**
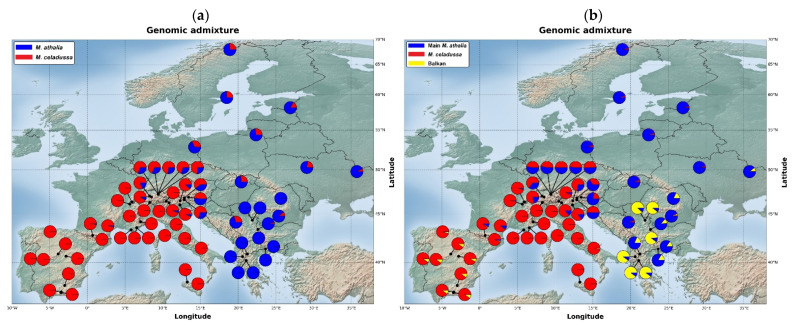
Pie chart map following STRUCTURE results of K = 2 (**a**) and K = 3 (**b**) based on ddRAD data, showing individual genomic admixture of *M. celadussa*–*M. athalia* according to geographic location.

The STRUCTURE analysis estimated the highest likelihood for the existence of two clusters in our dataset (Figure S5a). All STRUCTURE results from K = 2 to 4 (Figure S5b) showed frequent genomic admixture at the contact zone. However, the geographic distribution of genomic admixture within *M. athalia* varied depending on the K. At K = 2 admixture from *M*. *celadussa* extended into many of the analysed specimens of *M. athalia*, (all Northern and Eastern Europe) and to a lesser extent in the Balkans (Figure 3a). The pattern was different in results for K = 3 and K = 4 (Figure S5b), in which admixture between *M. celadussa*–main *M*. *athalia* was limited to the contact zone; in K = 3 there is also admixture in the Balkans, between the main *M*. *athalia* and the Balkan lineage, as well as slight admixture between Iberian *M. celadussa* and the Balkan lineage of *M. athalia*. (Figure 3b).

A PCoA visualization of the dissimilarity matrix (Figure 1a) confirmed this result since two main clusters were obtained (differentiated by PC2); the Balkan specimens as one cluster and a continuous group formed by main *M*. *athalia*–*M. celadussa* specimens as the other (which is spread along PC1). When plotted on the maps, the intermediate specimens between extreme *M. celadussa* and main *M. athalia* were located in the contact zone of the Alps (Figure 1b). Iberian *M. celadussa* was also recovered as a slightly differentiated group, thus confirming COI data (Figure 1a,b and Figure S6).

Analyses of genetic distance based on K = 2 clusters obtained F_st_ = 0.1967 and the diversity within populations was F_is_ = 0.6108. When applying a clustering model equivalent to morphological species, the values did not significantly change: F_st_ = 0.0964 and F_is_ = 0.6112, indicating that gene flow is generally high between and within populations.

### 3.6. Population History

**Figure 4:**
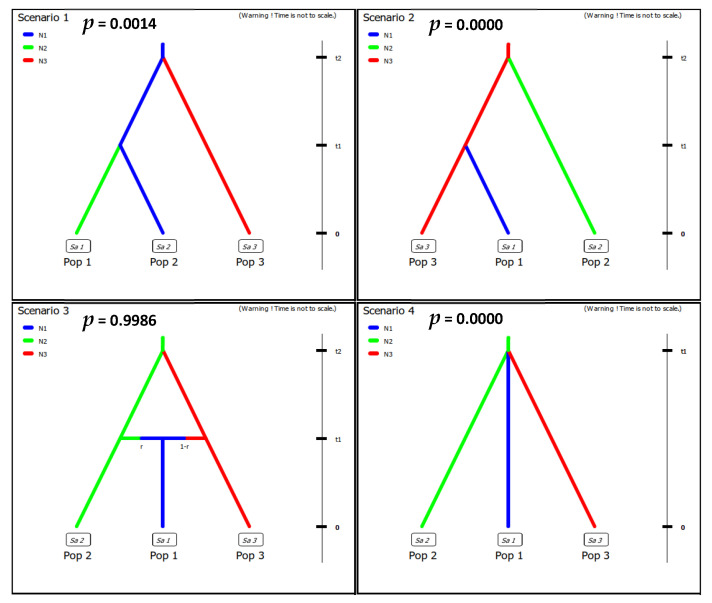
Demographic scenarios designed for DIYABC analysis. Pop 1: main *M. athalia*, Pop 2: Balkan lineage, Pop 3: *M. celadussa.* The logistic regressions of posterior probabilities are given for each scenario suggesting the third scenario as the fittest one.

Of four tested scenarios, scenario 3 had the highest logistic regression of posterior probability (0.9986 [0.9770, 1.0000]) with 2% of simulated data (Figure 4), which supports past hybridization. Testing for the model fitness based on principal component analysis confirmed that the admixture scenario fits well the empirical data because the observed data was located within the posterior probability distribution of the dataset (Table S4, Figure S7).

### 3.7. Isolation by Distance

**Figure 5:**
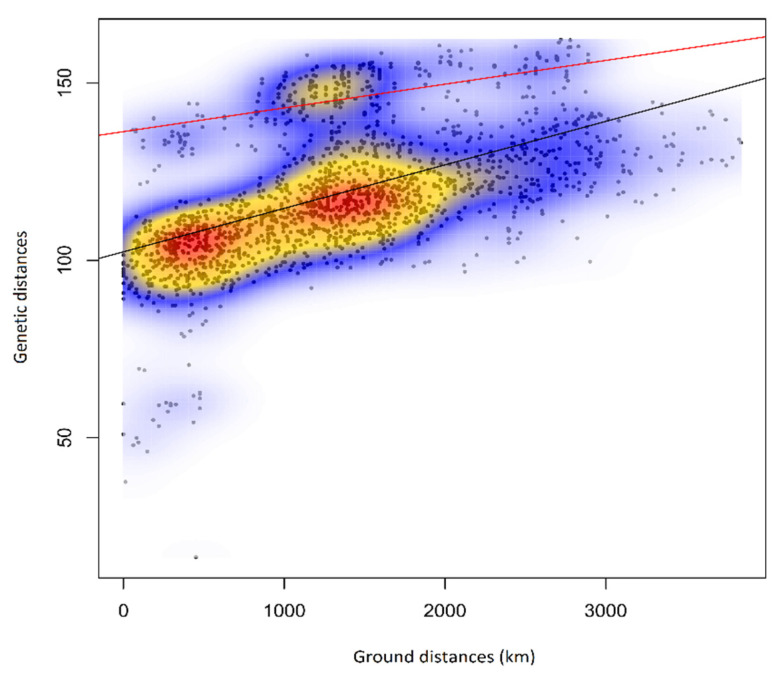
Isolation by distance plot illustrating the pattern of genetic differentiation in the *M. athalia*–*M. celadussa* species group (r^2^ = 0.536, *p* = 0.001) in relation to pairwise geographic distances. The black line represents the overall trend, while the red line represents the relationship between the Balkan clade and the group composed by main *M. athalia* + *M. celadussa*.

The isolation by distance (IBD) analysis for *M. athalia*–*M. celadussa* displayed one cloud of points indicating a continuous cline of genetic variation correlating with geographic distance, an exception being the Balkan lineage, displaying a higher level of differentiation given the geographic distance (smaller cloud above the main gene pool) (Figure 5). The Mantel test revealed a significant positive correlation of *r^2^ =* 0.504 (*p* = 0.001) between the two matrices indicating that genomic differentiation increases with geographical distance.

## 4. Discussion

*Melitaea athalia* and *M. celadussa* are among the most widespread and common European butterfly species [18,20,21,40,41]. However, their relationship has been the subject of considerable interest [15,16,42] due to their intermediate feature displays and occasional mito-morphological discordance at the contact zone. Here, we contributed a genome-wide assessment and interpreted results considering the spatial distribution of the observed diversity and the paleoclimatic reconstructions for Europe [43,44].

### 4.1. Genetic Structure and Levels of Admixture in M. athalia and M. celadussa

The topology of the ML tree based on ddRAD data largely reflects the pattern of COI differentiation, but only partially supports the reciprocal monophyly of *M. athalia* and *M. celadussa.* In the ddRAD tree, most of the *M. athalia* appeared as paraphyletic suggesting a certain degree of admixture with *M. celadussa*. Although ML phylogenetic reconstruction does not consider gene flow, the numerous potentially admixed specimens, especially from the contact zone, seem to generate the paraphyly (Figure 2).

Most specimens identified as *M. celadussa*, based on COI and genitalia, are also recovered as a monophyletic clade in the ML tree, with two exceptions showing mito-nuclear discordance in the area of contact (Figure 2). Mito-nuclear discordance is a relatively common phenomenon at contact zones, where it may be produced by events of hybridization [45]. The analyses of genomic structure and isolation by distance uncovered a continuum across the geographic range of *M. celadussa* and *M. athalia* due to the admixture at the contact zone (Alps). The degree of admixture declines to central and northern Europe, is at its lowest in the Balkans, and reflects a non-symmetric introgression north-east of the contact zone (Figure 3a) [46]. This could be an outcome of adaptive introgression of a set of *M. celadussa* genes into *M. athalia* eastward or, alternatively, the remnants of an ancient contact zone that may have been shifting to the west, being spatially established in the Alps e.g., [47]. The spread of strongly admixed individuals to the north in areas climatically available to these species only after the onset of the last interglacial could, in essence, reflect a natural phenomenon where recolonization happened by chance (founder effect). Alternatively, the current pattern could agree with the first scenario involving a selective advantage for admixed specimens and allowing for the fast introgression of selected genes, possibly through traits that are related to temperature, hibernation or changes in the larval host plants (hybrid fitness) [48,49,50,51]. Such a selection is likely to stabilize the geographic location of introgressed populations [52].

Several taxa of the genus *Melitaea* display notable levels of intraspecific genetic differentiation and have a challenging taxonomy [18,53]. We documented a well-differentiated clade in the Balkans that has only recently been reported based on DNA barcodes [18]. This lineage, with a long basal branch, is sister to the rest of the group for ddRAD data. An Approximate Bayesian Computations (ABC) analysis suggested a scenario indicating the hybrid nature for main European populations of *M. athalia* with regards to *M. celadussa* and the Balkan clade as more likely compared to scenarios with strict bifurcations among taxa (i.e., without gene flow) (Figure 4 and Figure S7, Table S4). However, it is still possible that the solution of ABC is only driven by the introgression between the main *M. athalia* and *M. celadussa* and that, as suggested by structure analyses with K = 3 (Figure 3b), and by comparing the ML tree with COI structure, the main *M. athalia* and the Balkan clade show only little genomic admixture. The dissimilarity matrix of genotype data (PCoA) and isolation by distance suggest that the Balkan lineage has a genetic component distinct from the *celadussa-athalia* group. Interestingly, samples of *M. athalia* representing the main genotypes have been collected almost syntopically with specimens representing the Balkan genotype without evidence of mito-nuclear discordance or strong genomic admixture. Further research with additional sampling is needed to better assess potential barriers to gene flow in the Balkans and to determine whether this lineage represents a distinct species [54,55].

### 4.2. Patterns of Variability in Male Genital Morphology

When the three variables measured for male genital morphology are combined in a PCA (Figure S4), two morphotypes clearly emerged corresponding to the genital variation described in previous studies [15,16]. Overall, morphometrics reveal a continuum of genital shape, but specimens with intermediate traits are only found in the contact zone (mainly in the Alps, but one instance in the Spanish Pyrenees, the western extreme of the putative contact zone), where also mito-nuclear discordance is found. Moreover, comparison of genital morphology and COI revealed cases of mito-morphological discordance, i.e., presence of mtDNA of *M. celadussa* in specimens with genital morphology of *M. athalia*. All cases of morphologically intermediate specimens and mito-morphologically mismatch specimens are attributed to *M. celadussa* by COI which suggests discordant mitochondrial and nuclear clines. In addition to stochastic reasons, sex-biased dispersal, assortative mating or asymmetric reproductive fitness can be among the causes for this discrepancy [56,57]. Similar cases of mito-morphological mismatch have been reported previously [45,55,58,59]. Possibly, a similar condition can be found all along the contact zone in central/southern France and reaching the Pyrenees [15,16,19] as suggested by one specimen from the Spanish Pyrenees (RVcoll08P221). Outside the contact zone, the populations do not display intermediate morphology, and genitalic measures for the three male specimens of the Balkan lineage fell within the variation of the typical *M. athalia*.

### 4.3. The Evolutionary Origin of the M. athalia–M. celadussa Distribution

Theoretically, secondary contact of diverged populations may result either in speciation through reinforcement or merging of the populations [60,61]. However, an unstable situation can be maintained at least for a certain time in which hybridization is limited to a narrow contact zone without the involvement of most populations. Under some circumstances, i.e., when hybrids have a lower fitness than pure individuals, populations close to the contact zone can behave as bi-stable systems that tend to be composed by individuals belonging to one species of the interacting pair [62]. Delayed sympatry and the creation of narrow contacts are relatively common phenomena in European butterflies [63,64,65] and mostly occur among closely related species because of reproductive interference and competition [66,67,68]. The mito-morphological discordance suggests that, despite the structural mismatch in genitalia, mating may result in successful sperm transfer and egg fertilization. Nevertheless, this phenomenon is restricted to a narrow contact zone where discordances between morphology, COI and nuclear markers can be found.

The divergence time estimated for *M. athalia* and *M. celadussa* (~2.2 Ma) [20] coincides nearly to onset of the Pleistocene glaciations (~2.6 Ma) and the repeated glacial cycles covering Northern Europe and most of the Alps with ice sheets have probably played a fundamental role in shaping the current distribution of these species. Presumably, the region was recently (re)colonized by *M. athalia and M. celadussa* post-glacially [18,69]. This evidence favours the secondary contact hypothesis, indicating that the initial differentiation likely happened in allopatry, although the process of differentiation may have included other contacts in previous interglacial periods. In the most probable scenario, the taxa examined here were fragmented into three main refugia in Europe along several glacial cycles: (i) Iberia and the Italian Peninsula for *M. celadussa*; (ii) the Balkan Peninsula for the Balkan lineage; and (iii) an eastern refugium for the main *M. athalia*, as suggested by the large distribution in Russia and Asia of this COI lineage (Figure S6). In agreement to this hypothesis, a recent study comparing the climatic niches of a series of European cryptic taxa showed that *M. athalia* has a climatic niche significantly shifted toward a colder and drier climate, typical of steppe areas, compared to *M. celadussa* [17]. At the end of the last glacial period, the Asiatic *M. athalia* could have been favoured in colonizing the temperate plains of central and northern Europe, reaching Scandinavia, due to the climatic similarity with its original habitat and, possibly, also due to a stronger propagule pressure. Concurring with the post-glacial range expansion, these populations of *M. athalia* came into secondary contact with *M. celadussa* and hybridized, although the existence of previously admixed populations cannot be ruled out. They also came into secondary contact with the Balkan lineage, apparently with a lower degree of hybridization despite morphological similarity, although here the limited sample size needs to be taken into account. Nevertheless, the rapid post-glacial expansion of the eastern *M. athalia* seems to have produced contact zones and blocked the expansion of the other two lineages out of their glacial refugia, namely *M. celadussa* in western Europe and the Balkan lineage in eastern Europe.

### 4.4. Species Delimitation

Morphological differentiation of genitalia often seems to have taken place along the process of speciation in European species pairs with highly similar external morphology [70,71,72], although cases having virtually indistinguishable genitalia are also known [70,73,74]. The biological species concept, in particular, largely relies on criteria that do not allow splitting populations into separate species until their genetic differentiation is significant enough to impede gene flow and consequent merging of populations [75]. On the other hand, natural hybridization between closely related species has been reported to occur in up to 10% of animal species [76] and in about 16% of European butterflies [77]. Hybridization and resulting introgression is particularly frequent between parapatric taxa. An increasing body of evidence suggests that introgression plays an important role in the process of speciation e.g., [63,78]. This observation led to the introduction of alternative definitions of the species concept. For example, the differential fitness concept identifies species as “groups of individuals that are reciprocally characterized by features that would have negative fitness effects in other groups and that cannot be regularly exchanged between groups upon contact” [79]. Therefore, critical questions to address are: (1) have the taxa developed barriers to gene flow, (2) if present, are hybrids having reduced fitness, and (3) what kind of patterns are expressed by other parapatric and sympatric European butterfly species?

According to our data, current hybridization is detected in the contact zone of main *M. athalia* and *M. celadussa.* While no mito-morphological discordance was detected outside the contact zone, STRUCTURE suggested widespread admixture in the nuclear genome of main *M. athalia* and *M. celadussa*, albeit to a lesser extent in the latter species for K = 2 and much lower admixture for K = 3. Should we therefore consider *M. athalia* and *M. celadussa* as valid at species or subspecies level, or consider them synonymous? By strictly relying on the reproductive isolation or on reciprocal phylogenetic monophyly, it would be hard to consider *M. celadussa* as a distinct species from *M. athalia*. On the other hand, the two taxa appear as distinct ecological entities (species) because the two main lineages are adapted to different climatic niches [17,80]. The answer is semantic in nature and depends upon the species concept applied. Whatever the solution over the species delimitation of parapatric and allopatric taxa will be, it is bound to be inherently arbitrary, for which reason more efforts should be made to find a consensus over the standards of delimitation of species under various evolutionary circumstances.

The Balkan lineage shows a different pattern. Despite the genetic divergence (Figure 2) and lack of introgression with main *M. athalia* (genetic structure at K = 2), we did not find differences in morphological traits in our samples. Moreover, due to the near sympatry with main *M. athalia*, their climatic niches likely largely overlap. While the potential drift could have played a role in the differentiation of the Balkan lineage, it might also represent another case of cryptic species among European butterflies. However, the sampling for this lineage is insufficient and this question will be addressed in a dedicated study.

## Data Availability

The *Melitaea* demultiplexed fastq data are archived in NCBI SRA: PRJNA638526. All 71 COI sequences are available in DS-MELITAEA on BOLD (dx.doi.org/10.5883/DS-MELITAEA) at https://www.boldsystems.org/ (accessed on 29 November 2021). The Jupyter cookbook script for Figure 3 is publicly available at https://doi.org/10.5281/zenodo.5762221 (accessed on 6 December 2021).

## Supplementary Materials

The following are available online at https://www.mdpi.com/article/10.3390/genes12122009/s1, Figure S1: Neighbour joining tree based on COI of specimens used in this study, Figure S2: Examples of male genitalia of *M. athalia* and *M. celadussa*, Figure S3: Scatterplot showing the relation between genitalic features measurements, Figure S4: PCA biplot showing the distribution of specimens and variables, Figure S5: STRUCTURE results, Figure S6: PCoA of COI data, Figure S7. The principal component analyses (PCAs) of summary statistics from DIYABC analysis, Table S1: *Melitaea* specimens used in this study, Table S2: Measurements of diagnostic male genitalia features in *M. athalia*-*M. celadussa*, Table S3: Summary statistics of ddRAD analysis in *M. athalia*–*M. celadussa*, Table S4: Posterior probability values of estimated parameters for the fittest scenario by DIYABC, Table S5: The specimens used for the COI analysis.
