# Supplementary material for: Genomics Reveal Admixture and Unexpected Patterns of Diversity in a Parapatric Pair of Butterflies
Source: Genes (Basel). 2021 Dec 17;12(12):2009. doi: 10.3390/genes12122009 (PMC8700966; doi:10.3390/genes12122009)
Supplement: Supplementary file 1 [file genes-12-02009-s001.zip › genes-1474068-suppl.pdf]

Genomics reveal admixture and unexpected patterns of diversity in a parapatric pair of butterflies

Tahami et al.

Supplementary material and methods

(a) Mitochondrial DNA data

We gathered COI sequences for all 71 specimens that were used for ddRADseq analyses (Table S1). For 62 of these specimens, sequences were generated by previous studies (1–3), while for nine specimens COI sequences were newly generated following standard protocols for DNA barcoding (e.g. 2). Sequences were aligned using Geneious Pro 6.1.8 created by Biomatters (http://www.geneious.com/). A Neighbor-joining (NJ) tree was built in MEGA X (4) and node supports were assessed based on 100 bootstrap replicates. The 71 COI sequences included four outgroup taxa: *M. britomartis*, 4 sequences; *M. aurelia*, 3 sequences; *M. deione*, 2 sequences; *M. caucasogenita/athalia*, 1 sequence. All 71 COI sequences are publicly available in DS-MELITAEA on BOLD (dx.doi.org/10.5883/DS-MELITAEA) at https://www.boldsystems.org/. It should be noted that *M. caucasogenita* is morphologically very similar to *M. athalia* and the distributions of the two taxa apparently overlap in the Caucasus (5). Because our specimen originates from the Caucasus, we tentatively called it *M. caucasogenita/athalia*. In our opinion, the evolutionary history of *M. caucasogenita* and its relationship with *M. athalia* requires further research.

In order to inspect the distribution of mtDNA lineages over the Palearctic, we added sequences of *M. athalia* and *M. celadussa* available in public repositories (BOLD, GenBank) thus obtaining a total dataset of 418 COI (Table S5). To visualize the genetic differentiation pattern over space, we calculated a p-distance dissimilarity matrix for COI and projected it in two dimensions by Principal Coordinate Analysis (PCoA) using the "cmdscale" R function. The resulting configuration for specimens was projected in the RGB colour space using the recluster R package (6) and individual RGB colours obtained by specimens were plotted on a map using pie charts.

(b) Morphological data

Genitalia morphometrics were performed for 42 male specimens of *M. athalia* and *M. celadussa* for which the abdomen was available to us. The genitalia were prepared following standard protocols (7). The distal part of the abdomen was placed into a 10% KOH solution and heated at 92 °C for 10 minutes. The abdomen was then placed in a petri dish and using a brush and forceps, the genitalia were isolated and cleaned, and were mounted on slides using Euparal. The genitalia were photographed in ventral view with a Leica DM1000 microscope and integrated Leica DF295 digital camera. Two to four photographs (depending on the quality of each sample) were stacked into single images using Zerene Stacker (zerenesystems.com/cms/home).

The genitalia terminology follows Higgins (8). Three elements of the male genitalia were measured (as in Figure S2) from stacked digital photographs using the software AxioVision 4.9.1: i) length of the sub-unci; ii) length of the posterior process of the valva; iii), the bifurcation on the posterior process of the valva, assessed based on four ordered values (0, absent; 1, small; 2, medium; 3, large) (Figure S2). We applied a Principal component analysis to the three variables by using the “dudi.mix” function of the ade4 R package (9) allowing the inclusion of continuous and ordered variables. To inspect the distribution of morphotypes and facilitate a direct comparison of the patterns among markers, we eliminated the effect of location and rotation between the bidimensional representations of COI PCoA and genitalia PCA with procrustes analysis, using the “recluster.procrustes” function of the recluster R package (6), which maximizes similarities among configurations on the basis of partially overlapping data sets. After procrustes, the bidimensional configuration for genitalia was projected in the RGB colour space and mapped as done for COI (see above).

(c) ddRAD library preparation

Genomic DNA was extracted using approximately two thirds of the thorax of either ethanol preserved or dry specimens. DNA extraction was done using the QIAGEN DNeasy Blood and Tissue kit following the manufacturer’s protocol. DNA extracts were gel-quantified visually in agarose 1% using the New England Biolabs 100 bp DNA Ladder (NEB). Due to the relatively low quality of initial genomic DNA, samples were subjected to whole genome amplification (WGA) following the instructions for 5 µl of template genomic DNA of REPLI-g® Mini Kits (QIAGEN). Afterwards, the concentration of amplified genomic DNA was quantified using the Quanti-iT Picogreen dsDNA reagents and kit (P7589, Invitrogen). The ddRAD tag

library was generated according to the same protocol described in Lee et al (10). Briefly, genomic DNA was double digested using the restriction enzyme pairs *Pst*I and *Mse*I and ligated to barcoded P1 and indexed P2 adapters, binding to *Pst*I and *Mse*I overhangs, respectively. DNA libraries were quantified using the High-Sensitivity DNA Analysis Kit in a 2100 Bioanalyzer (Agilent Technologies). The pooled library was sent for sequencing on an Illumina HiSeq 2500 PE 100 machine at FIMM (Institute for Molecular Medicine Finland). All obtained sequences are available at the NCBI Sequence Read Archive (SRA) [BioProject ID: PRJNA638526].

Table S1. *Melitaea* specimens used in this study.

Sample ID	BioSample accessions	Taxon	Country	Exact site	Lat	Lon
**RVcoll07E394**	SAMN15196501	*M. athalia*	Romania	Picior de Munte	44.8120	25.397
**RVcoll08M346**	SAMN15196502	*M. athalia*	Romania	1 Km E of Gheorgheni	46.743	25.664
**RVcoll10A789**	SAMN15196503	*M. athalia*	Estonia	2 km S. of Karilatsi, Polva County	58.1072	26.9178
**RVcoll12Z197**	SAMN15196504	*M. athalia*	Sweden	Riala, Norrtaelje municipality	59.612	18.485
**RVcoll13U296**	SAMN15196505	*M. athalia*	Italy	Val d`Oten	46.482	12.315
**RVcoll13U438**	SAMN15196506	*M. athalia*	Italy	Collina	46.589	12.853
**RVcoll14B773**	SAMN15196507	*M. athalia*	Albania	Voskopoje	40.592	20.596
**RVcoll14D059**	SAMN15196508	*M. athalia*	Bulgaria	Pamporovo, hotel Elitsa	41.624	24.701
**RVcoll14E853**	SAMN15196509	*M. athalia*	Serbia	Majdanpek, Debeli Lug	44.361	21.892
**RVcoll14E859**	SAMN15196510	*M. athalia*	Serbia	Majdanpek, Debeli Lug	44.361	21.892
**RVcoll14E904**	SAMN15196511	*M. athalia*	Serbia	Povlen Mt., near Pasna Ravan	44.156	19.693
**RVcoll14F303**	SAMN15196512	*M. athalia*	Serbia	Crni Vrh	43.392	22.612
**RVcoll14F407**	SAMN15196513	*M. athalia*	Bulgaria	Zemen, above the gorges	42.49	22.733
**RVcoll14F538**	SAMN15196514	*M. athalia*	Greece	Orvilos Mt.	41.371	23.633
**RVcoll14F650**	SAMN15196515	*M. athalia*	Greece	Pentalofos-Eptachori	40.205	21.064
**RVcoll14F666**	SAMN15196516	*M. athalia*	Greece	Pentalofos-Eptachori	40.205	21.064
**RVcoll14G434**	SAMN15196517	*M. athalia*	Greece	Agios Germanos	40.864	21.201
**RVcoll14V075**	SAMN15196518	*M. athalia*	Ukraine	Fedorovka, Novaya Vodolaga distr.	49.817	35.75
**RVcoll15I360**	SAMN15196519	*M. athalia*	Austria	Kohlmaier Hütte	46.8686	13.4262
**RVcoll15P033**	SAMN15196520	*M. athalia*	Ukraine	Korostyshiv	50.31	29.11
**RVcoll16H415**	SAMN15196521	*M. athalia*	Sweden	NW of Nikkaluokta (Cievrragorsa river valley)	67.8788	18.9024
**RVcoll16I052**	SAMN15196522	*M. athalia*	Poland	Siekierki	52.819	14.2346
**RVcoll16J000**	SAMN15196523	*M. athalia*	Slovakia	Plesivec	48.6139	20.4172
**RVcoll16J612**	SAMN15196524	*M. athalia*	Russia	Krasnolesye, Nesterovskiy rayon	54.3856	22.3677
**RVcoll11Y077**	SAMN15196525	*M. aurelia*	Italy	Pont d`Ael	44.436	8.402
**RVcoll12R765**	SAMN15196526	*M. aurelia*	Romania	Calarasi, padurea Ciornohal	47.620	27.230
**RVcoll15H019**	SAMN15196527	*M. aurelia*	Italy	Dego	45.678	7.221
**RVcoll08M100**	SAMN15196528	*M. britomatris*	Romania	Moldavia, Oraseni Vale (forest)	47.667	26.650
**RVcoll14E766**	SAMN15196529	*M. britomatris*	Romania	Capalnas	45.951	22.212
**RVcoll14N369**	SAMN15196530	*M. britomatris*	Ukraine	Nosivka district, Chernihiv region	50.980	31.780
**RVcoll14V076**	SAMN15196531	*M. britomatris*	Ukraine	Fedorovka, Novaya Vodolaga distr.	49.817	35.750
**ZFMK-TIS-8000434**	SAMN15196532	*M. caucasogenita /athalia*	Georgia	Samtskhe-Javakheti, Borjomi, 0.65km V of Kodiani,		
**RVcoll08H935**	SAMN15196533	*M. celadussa*	Spain	Hervas, Carretera Hervas-La Garganta	40.3240	-5.807

**RVcoll08J851**	SAMN15196534	*M. celadussa*	Spain	Posada de Valdeon	43.154	-4.92
**RVcoll08L852**	SAMN15196535	*M. celadussa*	Spain	Puerto de la Morcuera	40.878	-3.848
**RVcoll08M074**	SAMN15196536	*M. celadussa*	Spain	San Andres del Rey, La Alcarria	40.643	-2.814
**RVcoll08M915**	SAMN15196537	*M. celadussa*	Spain	Bassa d`Arres, Vall d`Aran	42.765	0.712
**RVcoll08P221**	SAMN15196538	*M. celadussa*	Spain	La Molina	42.354	1.951
**RVcoll11H561**	SAMN15196539	*M. celadussa*	Italy	San Martino alle Scale, Sicily	38.0950	13.2490
**RVcoll11H741**	SAMN15196540	*M. celadussa*	Italy	Portella Femmina Morta	37.92	14.66
**RVcoll11I433**	SAMN15196541	*M. celadussa*	Spain	3 km S Gueejar Sierra (road to Veleta, Sierra Nevada)	37.131	-3.447
**RVcoll11I507**	SAMN15196542	*M. celadussa*	Spain	SE of Cumbres Verdes (La Zubia)	37.083	-3.51
**RVcoll11I949**	SAMN15196543	*M. celadussa*	France	Chichilianne	44.81	5.585
**RVcoll12O623**	SAMN15196544	*M. celadussa*	France	Isola	44.201	7.074
**RVcoll12P926**	SAMN15196545	*M. celadussa*	France	Nans-les-Pins	43.352	5.826
**RVcoll12Q105**	SAMN15196546	*M. celadussa*	France	Callas	43.569	6.566
**RVcoll12Q106**	SAMN15196547	*M. celadussa*	France	Callas	43.569	6.566
**RVcoll13S845**	SAMN15196548	*M. celadussa*	Portugal	East of Manteigas	40.389	-7.534
**RVcoll13U092**	SAMN15196549	*M. celadussa*	Italy	Monte Terminillo	42.46	12.937
**RVcoll13U124**	SAMN15196550	*M. athalia*	Italy	Borgo Olivi	46.024	12.28
**RVcoll14E220**	SAMN15196551	*M. celadussa*	Spain	Cabanas al Nacimiento del Guadalquivir	37.767	-2.999
**RVcoll14J820**	SAMN15196552	*M. celadussa*	France	Belmont-sur-Rance	43.782	2.727
**RVcoll14L240**	SAMN15196553	*M. celadussa*	Italy	Campo Carlo Magno, Malga Vigo	46.263	10.836
**RVcoll15A654**	SAMN15196554	*M. celadussa*	Italy	Lodi	45.328	9.509
**RVcoll15A916**	SAMN15196555	*M. celadussa*	Italy	Campo nell Elba, Monumento	42.76	10.27
**RVcoll15G145**	SAMN15196556	*M. celadussa*	France	Septmoncel	46.3654	5.8983
**RVcoll15G841**	SAMN15196557	*M. celadussa*	Italy	Ausone	46.2936	8.27976
**RVcoll15I495**	SAMN15196558	*M. celadussa*	Austria	Wacht (Drau River)	46.7890	12.8747
**RVcoll15L146**	SAMN15196559	*M. celadussa*	Italy	Santa Caterina Valfurva	46.4240	10.4640
**Rvcoll15M133**	SAMN15196560	*M. celadussa*	France	Les Granges du Poizat	46.116	5.628
**RVcoll15N014**	SAMN15196561	*M. celadussa*	Italy	Alberona	41.449	15.112
**RVcoll16C754**	SAMN15196562	*M. celadussa*	Italy	Valbonella	43.924	11.792
**MAT-LU-K-122**	SAMN15196563	*M. celadussa*	Switzerland	Grüebli	47.0155	8.2525
**MAT-SG-W-135**	SAMN15196564	*M. celadussa*	Switzerland	Horbrunne	47.2075	9.3882
**MAT-SG-W-137**	SAMN15196565	*M. celadussa*	Switzerland	Horbrunne	47.2075	9.3882
**MAT-SG-W-138**	SAMN15196566	*M. celadussa*	Switzerland	Horbrunne	47.2075	9.3882
**MAT-SG-W-140**	SAMN15196567	*M. celadussa*	Switzerland	Horbrunne	47.2075	9.3882
**MAT-SG-W-144**	SAMN15196568	*M. athalia*	Switzerland	Horbrunne	47.2075	9.3882
**MAT-UR-I-146**	SAMN15196569	*M. celadussa*	Switzerland	Stettli	46.9072	8.5198
**RVcoll06A041**	SAMN15196570	*M. deione*	Spain	Vilamos, Vall d`Aran	36.9100	-3.2020
**RVcoll08M932**	SAMN15196571	*M. deione*	Spain	Barranco de los Lagartos, Cadiar	42.7430	0.7410

Table S2. Measurements of diagnostic male genitalia features in *M. athalia-M. celadussa*. Specimens were attributed to species based on both COI and morphology. The bifurcation of the posterior process of valva has been classified in four ordered categories (0, no bifurcation; 1, 2, 3, small, medium and large bifurcation, respectively).

Sample ID	Taxon (based on COI)	Taxon (based on genitalia)	Sub-unci (mm)	Posterior process of valva (mm)	Bifurcation of the posterior process of valva
**RVcoll08H935**	*M. celadussa*	*M. celadussa*	0.00	0.79	no
**RVcoll11H741**	*M. celadussa*	*M. celadussa*	0.00	0.68	no
**RVcoll11H561**	*M. celadussa*	*M. celadussa*	0.00	0.69	no
**RVcoll08M074**	*M. celadussa*	*M. celadussa*	0.00	0.68	no
**RVcoll15A654**	*M. celadussa*	*M. celadussa*	0.00	0.72	no
**RVcoll14E220**	*M. celadussa*	*M. celadussa*	0.00	0.72	no
**RVcoll12Q106**	*M. celadussa*	*M. celadussa*	0.00	0.62	no
**RVcoll12Q105**	*M. celadussa*	*M. celadussa*	0.00	0.64	no
**RVcoll12P926**	*M. celadussa*	*M. celadussa*	0.00	0.65	no
**RVcoll16C754**	*M. celadussa*	*M. celadussa*	0.00	0.69	no
**RVcoll15N014**	*M. celadussa*	*M. celadussa*	0.00	0.65	no
**RVcoll15G841**	*M. celadussa*	*M. celadussa*	0.00	0.64	no
**RVcoll15A916**	*M. celadussa*	*M. celadussa*	0.00	0.77	no
**RVcoll08L852**	*M. celadussa*	*M. celadussa*	0.03	0.64	no
**RVcoll08M915**	*M. celadussa*	*M. celadussa*	0.04	0.67	no
**RVcoll13U092**	*M. celadussa*	*M. celadussa*	0.08	0.65	no
**RVcoll15G145**	*M. celadussa*	intermediate	0.13	0.68	no
**MAT-SG-W-138**	*M. celadussa*	intermediate	0.13	0.62	yes, small
**RVcoll08P221**	*M. celadussa*	intermediate	0.15	0.78	no
**RVcoll11I949**	*M. celadussa*	intermediate	0.19	0.67	no
**MAT-LU-K-122**	*M. celadussa*	intermediate	0.20	0.65	no
**MAT-UR-I-146**	*M. celadussa*	intermediate	0.21	0.67	no
**RVcoll14F303**	*M. athalia*	*M. athalia*	0.26	0.60	yes, large
**MAT-SG-W-135**	*M. celadussa*	*M. athalia*	0.26	0.63	yes, medium
**MAT-SG-W-140**	*M. celadussa*	*M. athalia*	0.27	0.65	yes, small
**RVcoll16I052**	*M. athalia*	*M. athalia*	0.28	0.52	yes, medium
**RVcoll16H415**	*M. athalia*	*M. athalia*	0.28	0.52	yes, large
**RVcoll14F666**	Balkan	*M. athalia*	0.28	0.45	yes, large
**RVcoll16J000**	*M. athalia*	*M. athalia*	0.29	0.53	yes, large
**RVcoll14F650**	Balkan	*M. athalia*	0.29	0.57	yes, small
**RVcoll15I360**	*M. athalia*	*M. athalia*	0.30	0.60	yes, large
**RVcoll14V075**	*M. athalia*	*M. athalia*	0.31	0.59	yes, large
**RVcoll12Z197**	*M. athalia*	*M. athalia*	0.32	0.46	yes, large
**RVcoll14E859**	Balkan	*M. athalia*	0.32	0.52	yes, large
**MAT-SG-W-137**	*M. celadussa*	*M. athalia*	0.32	0.58	yes, medium
**RVcoll10A789**	*M. athalia*	*M. athalia*	0.34	0.59	yes, large
**MAT-SG-W-144**	*M. celadussa*	*M. athalia*	0.34	0.64	yes, large
**RVcoll14F538**	*M. athalia*	*M. athalia*	0.34	0.68	yes, large
**RVcoll13U438**	*M. athalia*	*M. athalia*	0.35	0.56	yes, medium

**RVcoll14L240**	*M. celadussa*	*M. athalia*	0.36	0.74	yes, small
**RVcoll13U124**	*M. celadussa*	*M. athalia*	0.37	0.77	yes, small
**RVcoll13U296**	*M. athalia*	*M. athalia*	0.41	0.73	yes, large

Table S3. Summary statistics of ddRAD analysis in *M. athalia*−*M. celadussa* using the reference assembly method in ipyrad-0.9.31.

Sample ID	Raw reads	Reads passed filter	Clusters at 85%	Loci retained in assembly	Sample coverage (%)
**RVcoll07E394**	4673782	4673359	14500	4180	11.14
**RVcoll08M346**	1180105	1180010	21709	5737	15.30
**RVcoll10A789**	1362089	1361958	18140	5075	13.53
**RVcoll12Z197**	3428846	3428520	26985	7994	21.31
**RVcoll13U296**	2349154	2348944	16355	5609	14.95
**RVcoll13U438**	1794097	1793939	12193	3557	9.48
**RVcoll14B773**	1334180	1334062	22537	5597	14.92
**RVcoll14D059**	2904690	2904422	22346	5568	14.85
**RVcoll14E853**	1735363	1735214	16089	4763	12.70
**RVcoll14E859**	1336558	1336439	14023	4374	11.66
**RVcoll14E904**	312335	312305	11555	3538	9.43
**RVcoll14F303**	514177	514133	11188	3875	10.33
**RVcoll14F407**	526337	526291	12533	3256	8.68
**RVcoll14F538**	1418876	1418752	16369	4439	11.84
**RVcoll14F650**	800953	800882	14182	3939	10.50
**RVcoll14F666**	899231	899141	14859	3352	8.94
**RVcoll14G434**	822465	822392	30231	5943	15.85
**RVcoll14V075**	2639967	2639743	24563	7873	20.99
**RVcoll15I360**	3447665	3447314	13995	4106	10.95
**RVcoll15P033**	2394804	2394573	21683	7043	18.78
**RVcoll16H415**	6631017	6630426	18917	6500	17.33
**RVcoll16I052**	1399866	1399738	18588	5416	14.44
**RVcoll16J000**	1669413	1669269	18005	5315	14.17
**RVcoll16J612**	3478095	3477748	18272	5091	13.57
**RVcoll15H019**	2936423	2936146	22346	5093	13.58
**RVcoll12R765**	753456	753380	20325	4568	12.18
**RVcoll11Y077**	3364094	3363784	18608	4633	12.35
**RVcoll08M100**	814284	814187	19502	4749	12.66
**RVcoll14E766**	1920916	1920734	14033	4000	10.66
**RVcoll14N369**	708815	708744	13768	3456	9.21
**RVcoll14V076**	1953329	1953126	18872	4978	13.27
**ZFMK-TIS-8000434**	2035030	2034813	20384	4745	12.65
**RVcoll08H935**	678809	678752	12296	2681	7.15
**RVcoll08J851**	2139413	2139191	18332	5194	13.85
**RVcoll08L852**	1162267	1162161	20459	5205	13.88
**RVcoll08M074**	1233917	1233798	15949	4903	13.07
**RVcoll08M915**	1320091	1319987	28081	6346	16.92
**RVcoll08P221**	2362060	2361835	26702	6765	18.04
**RVcoll11H561**	912690	912613	13419	3955	10.54
**RVcoll11H741**	2128167	2127974	16075	4558	12.15
**RVcoll11I433**	4085104	4084685	17209	4894	13.05
**RVcoll11I507**	1682980	1682848	13272	4070	10.85

**RVcoll11I949**	1087324	1087215	17448	5707	15.22
**RVcoll12O623**	1844190	1844014	15066	4894	13.05
**RVcoll12P926**	1807939	1807757	17564	4403	11.74
**RVcoll12Q105**	1903944	1903771	15983	4940	13.17
**RVcoll12Q106**	782535	782461	14671	4191	11.17
**RVcoll13S845**	4178603	4178173	15961	3919	10.45
**RVcoll13U092**	2365351	2365119	16590	3899	10.40
**RVcoll13U124**	1047273	1047162	14570	4658	12.42
**RVcoll14E220**	5057710	5057299	10992	2454	6.54
**RVcoll14J820**	2919802	2919525	23287	6539	17.43
**RVcoll14L240**	2293920	2293724	17637	5047	13.46
**RVcoll15A654**	684166	684105	12276	2336	6.23
**RVcoll15A916**	442919	442886	14948	2934	7.82
**RVcoll15G145**	1099739	1099637	16438	4704	12.54
**RVcoll15G841**	971153	971078	26138	6134	16.35
**RVcoll15I495**	1365715	1365567	14571	4710	12.56
**RVcoll15L146**	273904	273881	14653	3820	10.18
**Rvcoll15M133**	2963178	2962880	21183	5808	15.49
**RVcoll15N014**	2912245	2911962	24242	5083	13.55
**RVcoll16C754**	818903	818831	21637	5107	13.62
**MAT-LU-K-122**	1511382	1511226	29260	7416	19.77
**MAT-SG-W-135**	1630628	1630488	32584	8032	21.41
**MAT-SG-W-137**	863921	863860	21263	5749	15.33
**MAT-SG-W-138**	1308911	1308809	13360	3914	10.44
**MAT-SG-W-140**	489972	489925	11706	3800	10.13
**MAT-SG-W-144**	2207497	2207274	33001	9051	24.13
**MAT-UR-I-146**	1481659	1481541	22100	5116	13.64
**RVcoll08M932**	1254596	1254478	22563	4917	13.11
**RVcoll06A041**	3118650	3118338	11197	3091	8.24

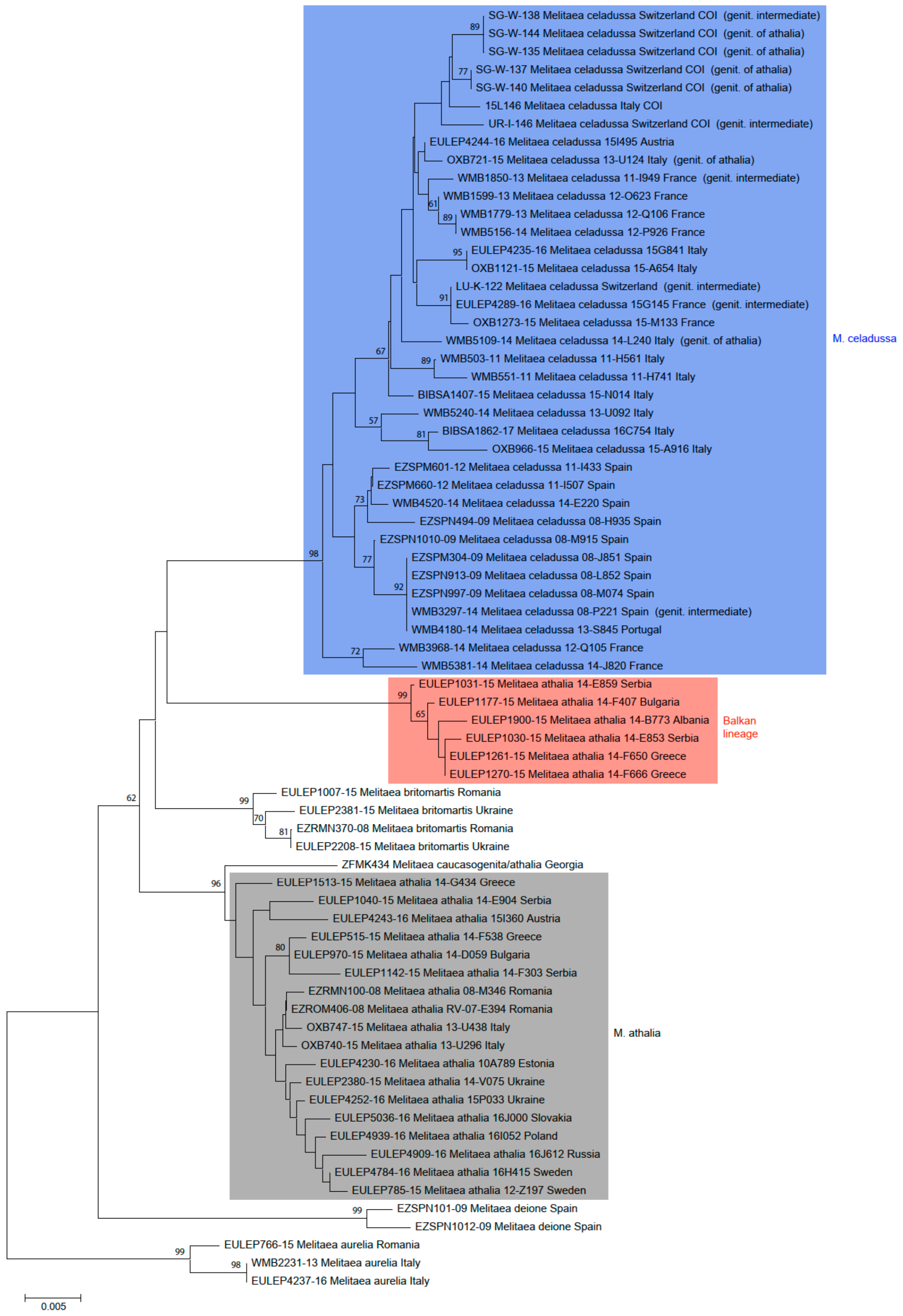

Figure S1. Neighbor joining tree based on COI of specimens used in this study. Bootstrap supports (> 50) are indicated next to recovered nodes.

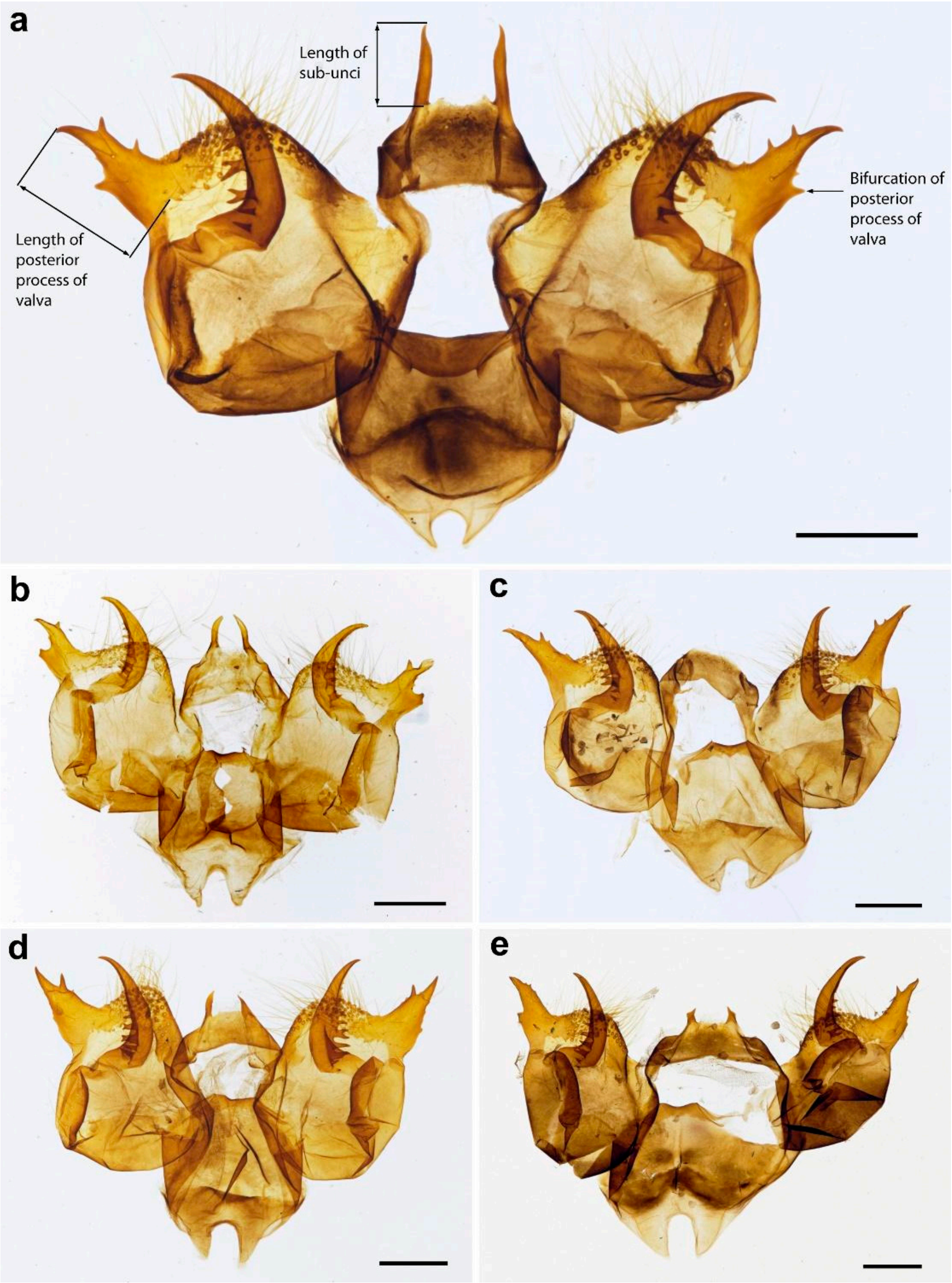

Figure S2. Examples of male genitalia of *Melitaea athalia* and *M. celadussa*. **a.** *M. athalia*, RVcoll13U438, Italy. Measured/assessed elements of the genitalia are indicated; **b.** *M. athalia* (Balkan lineage), RVcoll14F666, Greece; **c.** *M. celadussa*, RVcoll15N014, Italy; **d.** Specimen with intermediate characters between *M. athalia* and *M. celadussa*, LUK122, Switzerland; **e.** Specimen with intermediate characters between *M. athalia* and *M. celadussa*, SGW138, Switzerland. Scale bars represent 0.5 mm.

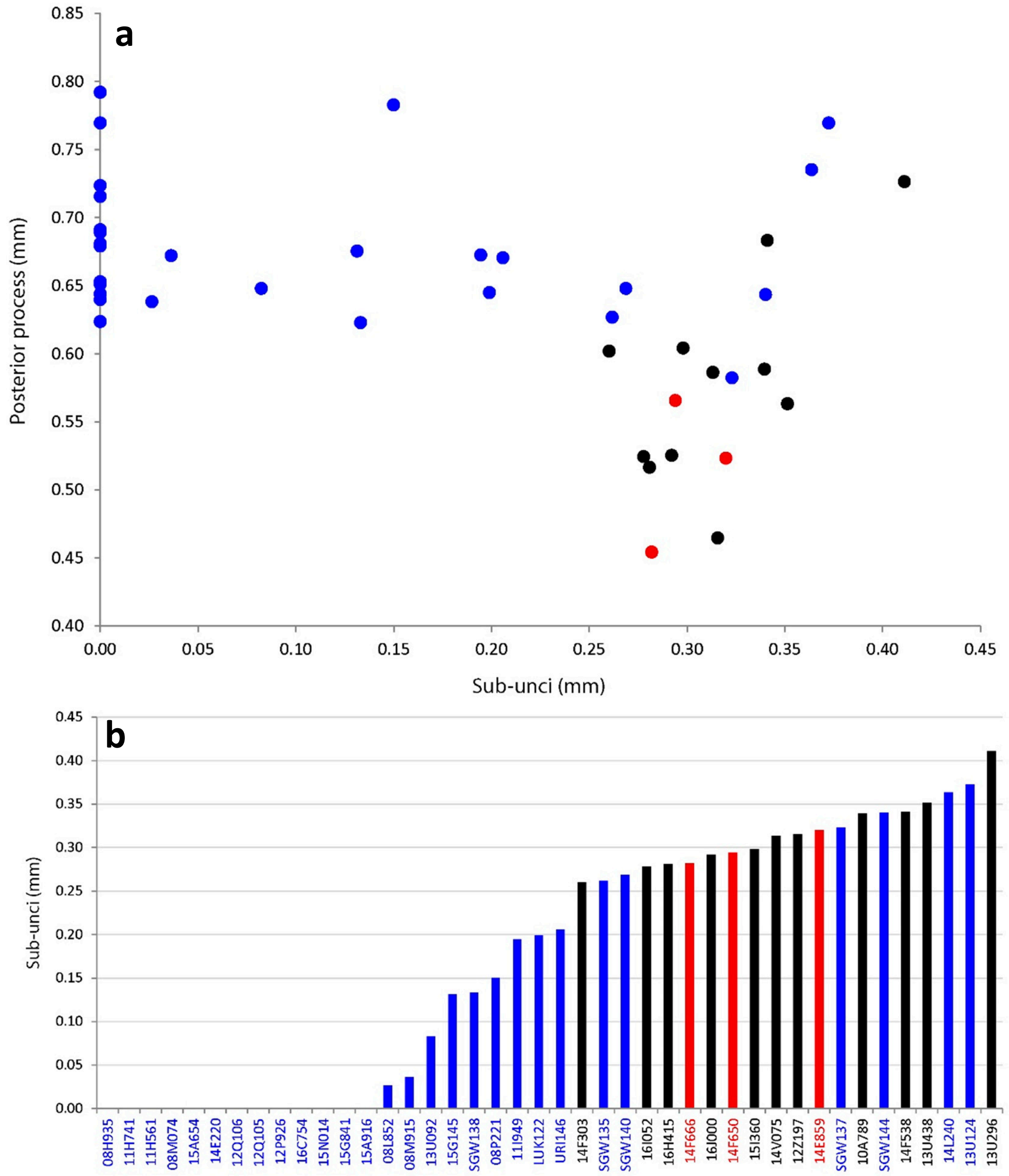

Figure S3. **a.** Scatterplot showing the relation between the length of sub-unci and the posterior process of valva in *M. athalia-M. celadussa*. **b.** Male genitalia measurements of sub-unci length. Black indicates *M. athalia*, blue indicates *M. celadussa* and red indicates specimens from the Balkan clade. Taxa are attributed based on COI.

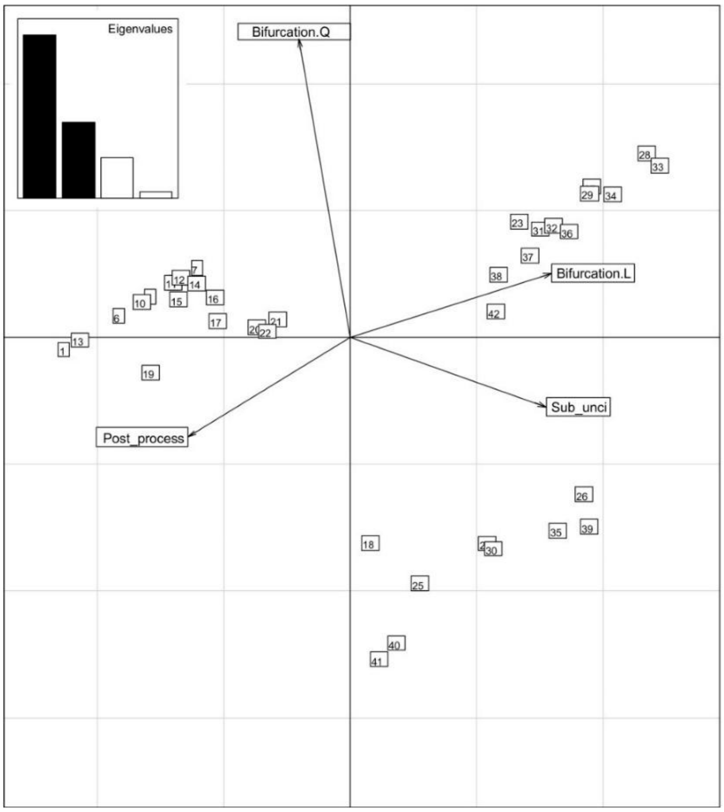

Figure S4. PCA biplot showing the distribution of specimens and variables. The *M. celadussa* morphotype is clustered in the left (no bifurcation and short sub-unci) and the *M. athalia* morphotype appears to the right (small, medium and large bifurcation, and long sub-unci).

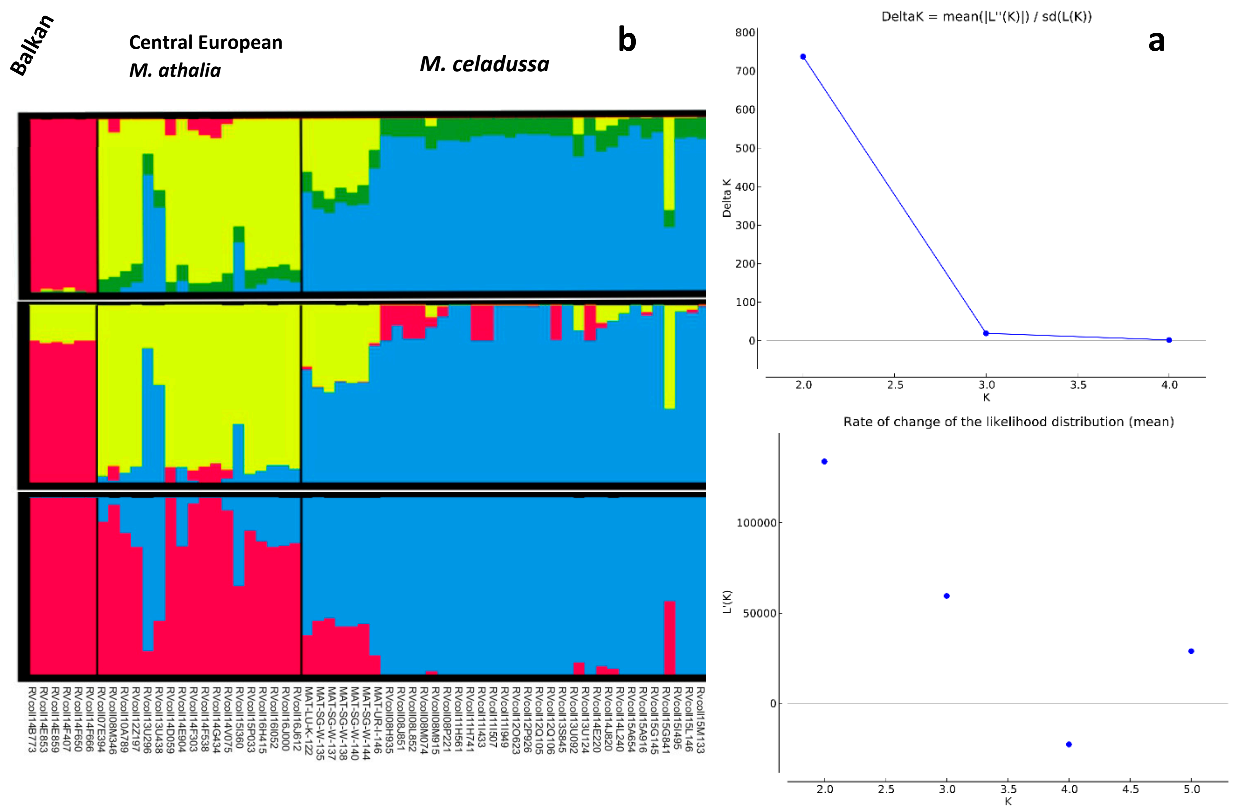

Figure S5. **a.** Genomic structural analysis of *M. athalia*-*M. celadussa* for ΔK=5 showing the maximum value at K=2. **b.** The aligned maps show cluster assignments at 2-4, from bottom to top.

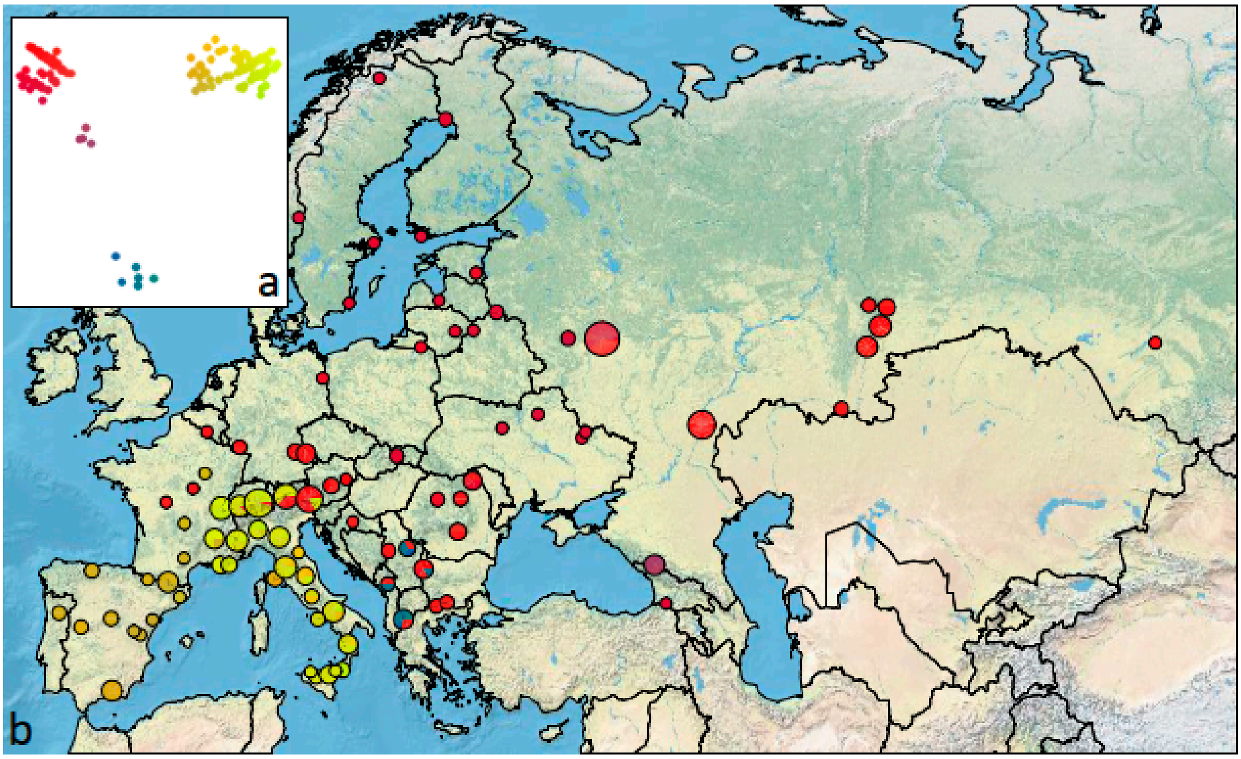

Figure S6. **a.** The configuration obtained after PCoA of COI distances projected in the RGB space and **b.** the location of each specimen on the map

Table S4. Posterior probability values of estimated parameters for the fittest scenario (sc3) by DIYABC v.2.1.0. based on 1% of the closest simulated data sets (scaled). N = effective population size, t = divergence time.

Parameter	mean	median	mode	2.5% CI	97.5% CI
**N1**	8.45E-01	4.90E-01	4.16E-01	4.16E-01	2.69E+00
**N2**	3.09E-01	5.30E-03	4.06E-03	4.06E-03	1.96E+00
**N3**	8.72E-03	7.81E-03	7.81E-03	7.81E-03	7.81E-03
**t1**	2.51E-01	1.62E-01	5.36E-02	1.28E-02	1.26E+00
**t2**	1.55E-01	1.54E-01	1.54E-01	1.54E-01	1.54E-01

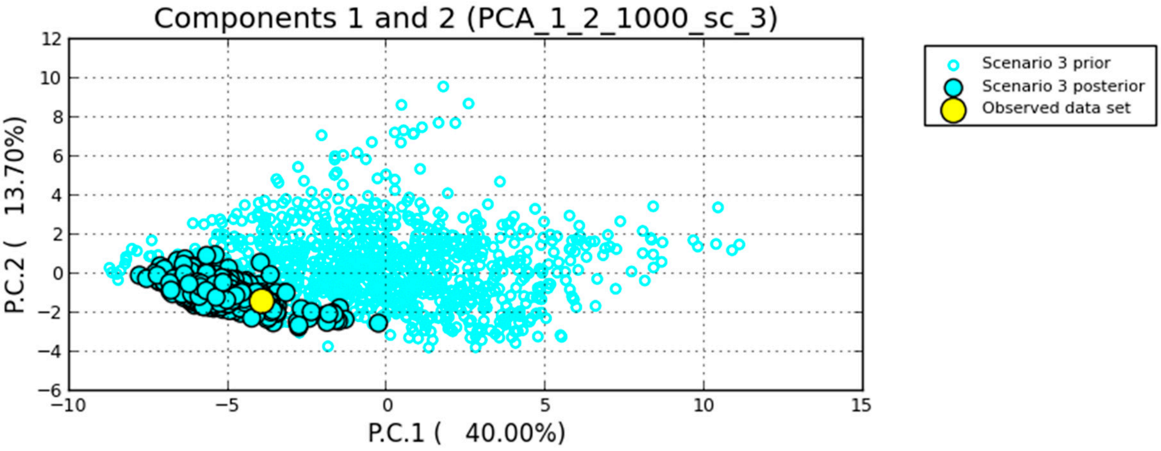

Figure S7. The principal component analyses (PCAs) of summary statistics were performed using DIYABC v.2.1.0 software for the prevailing admixture scenario based on the computer simulation of SNPs data.

Table S5. The specimens used for the COI analysis. Identifications are based on COI. Country of origin, decimal degrees coordinates, as well as BOLD and GenBank codes are indicated.

Sample.ID	Species	Country	Latitude	Longitude	BOLD ID	Genbank code
RVcoll09V134	*Melitaea celadussa*	Spain	36.964	-2.855	EZSPC1423-10	KP870439
RVcoll09V135	*Melitaea celadussa*	Spain	36.987	-3.262	EZSPC1424-10	HM901538
RVcoll09V136	*Melitaea celadussa*	Spain	36.987	-3.262	EZSPC1425-10	HM901539
RVcoll09V137	*Melitaea celadussa*	Spain	36.987	-3.262	EZSPC1426-10	HM901540
RVcoll11I507	*Melitaea celadussa*	Spain	37.083	-3.51	EZSPM660-12	KP870246
RVcoll08J954	*Melitaea celadussa*	Spain	37.107	-3.392	EZSPC1056-10	HM901259
RVcoll11I433	*Melitaea celadussa*	Spain	37.131	-3.447	EZSPM601-12	KP871044
RVcoll14E220	*Melitaea celadussa*	Spain	37.767	-2.999	WMB4520-14	NA
RVcoll11H980	*Melitaea celadussa*	Italy	37.8	13.99	BIBSA1747-16	NA
RVcoll11H749	*Melitaea celadussa*	Italy	37.85	14.71	WMB556-11	NA
RVcoll10C638	*Melitaea celadussa*	Italy	37.868	13.384	WMB2877-14	NA
RVcoll11H741	*Melitaea celadussa*	Italy	37.92	14.66	WMB551-11	NA
RVcoll12R009	*Melitaea celadussa*	Italy	37.967	14.75	WMB2575-13	NA
RVcoll11H561	*Melitaea celadussa*	Italy	38.08	13.25	WMB503-11	NA
RVcoll10C670	*Melitaea celadussa*	Italy	38.084	15.834	WMB2295-13	NA
RVcollLD0251	*Melitaea celadussa*	Italy	38.135	15.285	OXB1029-15	NA
RVcoll11I120	*Melitaea celadussa*	Italy	38.201	15.98	WMB3854-14	NA
RVcoll11I170	*Melitaea celadussa*	Italy	38.24	15.71	WMB3857-14	NA
RVcoll10C542	*Melitaea celadussa*	Italy	38.372	16.236	WMB2286-13	NA
LEPSS00382	*Melitaea celadussa*	Italy	39.3881	16.5578	BIBSA1554-16	NA
LEPSS00090	*Melitaea celadussa*	Italy	39.3889	16.6022	BIBSA090-14	NA
LEPSS00114	*Melitaea celadussa*	Italy	39.3917	16.5303	BIBSA494-15	NA
RVcoll11I247	*Melitaea celadussa*	Italy	39.86	16.07	BIBSA1744-16	NA
RVcoll220602AM3	*Melitaea celadussa*	Spain	39.925	-0.879	EZSPC1273-10	JN274545

RVcoll11I207	*Melitaea celadussa*	Italy	39.93	16.15	BIBSA679-15	NA
RVcoll11I204	*Melitaea celadussa*	Italy	39.94	16.15	WMB651-11	NA
RVcoll100908AW26	*Melitaea celadussa*	Spain	40.126	-1.432	EZSPC1298-10	HM901470
GWORU094-10	*Melitaea celadussa*	Italy	40.1636	15.9003	GWORU094-10	HM910514-SUPPRESSED
RVcoll14F650	*Melitaea athalia*	Greece	40.205	21.064	EULEP1261-15	NA
RVcoll14F666	*Melitaea athalia*	Greece	40.205	21.064	EULEP1270-15	NA
RVcoll08H904	*Melitaea celadussa*	Spain	40.324	-5.807	EZSPN473-09	GU676878
RVcoll08H935	*Melitaea celadussa*	Spain	40.324	-5.807	EZSPN494-09	GU676899
RVcoll17G017	*Melitaea athalia*	Albania	40.34	20.67	EULEP5918-18	NA
RVcoll13S845	*Melitaea celadussa*	Portugal	40.389	-7.534	WMB4180-14	NA
RVcoll14B773	*Melitaea athalia*	Albania	40.592	20.596	EULEP1900-15	NA
RVcoll08M074	*Melitaea celadussa*	Spain	40.643	-2.814	EZSPN997-09	GU676161
RVcoll14I410	*Melitaea celadussa*	Italy	40.69	14.996	WMB4863-14	NA
RVcoll210907WR65	*Melitaea celadussa*	Spain	40.698	0.101	EZSPC1269-10	HM901454
RVcollLD2631	*Melitaea celadussa*	Italy	40.729	13.834	WMB3098-14	NA
RVcollLD2632	*Melitaea celadussa*	Italy	40.729	13.834	WMB3099-14	NA
RVcoll14O121	*Melitaea athalia*	Greece	40.79	21.32	EULEP2988-15	NA
RVcoll14I434	*Melitaea celadussa*	Italy	40.818	15.097	WMB4887-14	NA
RVcoll14I426	*Melitaea celadussa*	Italy	40.834	15.071	WMB4879-14	NA
RVcoll14G434	*Melitaea athalia*	Greece	40.864	21.201	EULEP1513-15	NA
RVcoll08L852	*Melitaea celadussa*	Spain	40.878	-3.848	EZSPN913-09	GU676244
RVcoll15C002	*Melitaea celadussa*	Italy	40.96	15.595	BIBSA990-15	NA
RVcoll14F538	*Melitaea athalia*	Greece	41.371	23.633	EULEP515-15	NA
RVcoll14F546	*Melitaea athalia*	Greece	41.371	23.633	EULEP1234-15	NA
RVcoll15N014	*Melitaea celadussa*	Italy	41.449	15.112	BIBSA1407-15	NA
RVcoll15M836	*Melitaea celadussa*	Italy	41.457	14.382	BIBSA1343-15	NA
RVcoll15M840	*Melitaea celadussa*	Italy	41.457	14.382	BIBSA1346-15	NA

RVcoll14C353	*Melitaea athalia*	Greece	41.504	24.4	EULEP869-15	NA
RVcoll15M960	*Melitaea celadussa*	Italy	41.523	14.612	BIBSA1379-15	NA
RVcoll14D059	*Melitaea athalia*	Bulgaria	41.624	24.701	EULEP970-15	NA
RVcoll08J143	*Melitaea celadussa*	Portugal	41.682	-7.71	EZSPN583-09	GU676562
RVcoll13S715	*Melitaea celadussa*	Italy	41.748	15.998	WMB4158-14	NA
RVcoll13S742	*Melitaea celadussa*	Italy	41.82	13.32	WMB4170-14	NA
RVcoll07Z072	*Melitaea celadussa*	Spain	41.833	2.367	EZSPC540-09	GU669727
RVcoll14I494	*Melitaea celadussa*	Italy	41.908	13.343	WMB4947-14	NA
RVcoll17G015	*Melitaea athalia*	Albania	41.98	20.58	EULEP5916-18	NA
RVcoll08P221	*Melitaea celadussa*	Spain	42.354	1.951	WMB3297-14	NA
RVcoll14U528	*Melitaea athalia*	Albania	42.37268	19.57273	EULEP5829-18	NA
RVcoll12Q707	*Melitaea celadussa*	Italy	42.4	13.767	WMB2028-13	NA
RVcoll07C635	*Melitaea celadussa*	Spain	42.448	1.781	EZSPC539-09	GU669726
RVcoll07C680	*Melitaea celadussa*	Spain	42.448	1.781	EZSPC560-09	GU669707
RVcoll13U092	*Melitaea celadussa*	Italy	42.46	12.937	WMB5240-14	NA
RVcoll07C071	*Melitaea celadussa*	Spain	42.474	1.933	EZROM734-08	KP870776
RVcoll08P214	*Melitaea celadussa*	France	42.488	1.856	EZSPM027-09	GU676006
RVcoll14F407	*Melitaea athalia*	Bulgaria	42.49	22.733	EULEP1177-15	NA
RVcoll12Z356	*Melitaea celadussa*	Spain	42.7	0.87	WMB4040-14	NA
RVcoll070611MH09	*Melitaea celadussa*	Spain	42.71	-0.29	WMB4553-14	NA
RVcoll15A914	*Melitaea celadussa*	Italy	42.76	10.27	OXB964-15	NA
RVcoll15A915	*Melitaea celadussa*	Italy	42.76	10.27	OXB965-15	NA
RVcoll15A916	*Melitaea celadussa*	Italy	42.76	10.27	OXB966-15	NA
RVcoll08M915	*Melitaea celadussa*	Spain	42.765	0.712	EZSPN1010-09	GU676148
RVcoll08P394	*Melitaea celadussa*	Spain	42.776	0.832	EZSPC207-09	KP870866
RVcoll08P395	*Melitaea celadussa*	Spain	42.776	0.832	EZSPC257-09	KP870367
RVcoll14O127	*Melitaea celadussa*	Italy	42.954	13.211	EULEP2994-15	NA

RVcoll12R426	*Melitaea celadussa*	Italy	42.958	10.535	BIBSA958-15	NA
RVcollLD2686	*Melitaea celadussa*	Italy	42.968	10.531	WMB4686-14	NA
RVcoll15N097	*Melitaea celadussa*	Italy	43.05	10.62	OXB1143-15	NA
RVcoll15N098	*Melitaea celadussa*	Italy	43.05	10.62	OXB1144-15	NA
RVcoll12R404	*Melitaea celadussa*	Italy	43.081	11.169	BIBSA957-15	NA
RVcoll10C728	*Melitaea celadussa*	Italy	43.136	11.56	WMB1082-13	NA
RVcoll08J851	*Melitaea celadussa*	Spain	43.154	-4.92	EZSPM304-09	GU675930
RVcoll16L393	*Melitaea celadussa*	Spain	43.15631	-4.91212	NA	NA
RVcoll12Q078	*Melitaea celadussa*	France	43.237	6.334	WMB1775-13	NA
RVcoll12P876	*Melitaea celadussa*	France	43.349	5.73	WMB1727-13	NA
RVcoll12P926	*Melitaea celadussa*	France	43.352	5.826	WMB5156-14	NA
RVcoll14F060	*Melitaea athalia*	Serbia	43.368	22.594	EULEP514-15	NA
RVcoll14F120	*Melitaea athalia*	Serbia	43.386	22.592	EULEP1096-15	NA
RVcoll14F303	*Melitaea athalia*	Serbia	43.392	22.612	EULEP1142-15	NA
RVcoll14F327	*Melitaea athalia*	Serbia	43.392	22.612	EULEP1149-15	NA
RVcoll14F216	*Melitaea athalia*	Serbia	43.396	22.368	EULEP1122-15	NA
RVcoll15N099	*Melitaea celadussa*	Italy	43.48	11.835	OXB1145-15	NA
RVcoll15A927	*Melitaea celadussa*	Italy	43.501	10.451	OXB977-15	NA
RVcoll10B642	*Melitaea celadussa*	France	43.554	5.73	WMB3486-14	NA
RVcoll10B644	*Melitaea celadussa*	France	43.554	5.73	WMB111-11	NA
RVcoll12Q105	*Melitaea celadussa*	France	43.569	6.566	WMB3968-14	NA
RVcoll12Q106	*Melitaea celadussa*	France	43.569	6.566	WMB1779-13	NA
RVcoll16C719	*Melitaea celadussa*	Italy	43.78	11.665	BIBSA1842-17	NA
RVcoll14J820	*Melitaea celadussa*	France	43.782	2.727	WMB5381-14	NA
RVcoll19C206	*Melitaea celadussa*	Italy	43.814	11.767	BIBSA2059-19	NA
RVcoll16C754	*Melitaea celadussa*	Italy	43.924	11.792	BIBSA1862-17	NA
RVcoll09X863	*Melitaea celadussa*	Italy	44.063	10.807	BIBSA951-15	NA

RVcoll14L226	*Melitaea celadussa*	Italy	44.078	12.206	WMB5095-14	NA
RVcoll14V142	*Melitaea celadussa*	Italy	44.1021	10.2326	EULEP4253-16	NA
RVcoll15N100	*Melitaea celadussa*	Italy	44.116	11.236	OXB1146-15	NA
RVcoll14E989	*Melitaea athalia*	Serbia	44.122	20.015	EULEP1070-15	NA
RVcoll14A525	*Melitaea celadussa*	Italy	44.125	10.777	WMB4317-14	NA
RVcoll14E904	*Melitaea athalia*	Serbia	44.156	19.693	EULEP1040-15	NA
RVcoll14E908	*Melitaea athalia*	Serbia	44.156	19.693	EULEP1042-15	NA
RVcoll14E043	*Melitaea celadussa*	Italy	44.186	7.271	BIBSA247-15	NA
RVcoll12O623	*Melitaea celadussa*	France	44.201	7.074	WMB1599-13	NA
RVcoll12O624	*Melitaea celadussa*	France	44.201	7.074	WMB3799-14	NA
RVcoll14E853	*Melitaea athalia*	Serbia	44.361	21.892	EULEP1030-15	NA
RVcoll14E859	*Melitaea athalia*	Serbia	44.361	21.892	EULEP1031-15	NA
OXBTGS1313	*Melitaea celadussa*	France	44.4866	5.38041	OXB1595-16	NA
RVcoll14I538	*Melitaea celadussa*	Italy	44.487	9.497	WMB4991-14	NA
RVcoll14D573	*Melitaea celadussa*	Italy	44.52	8.7	BIBSA124-15	NA
OXBTGS1289	*Melitaea celadussa*	France	44.534	5.37034	OXB1571-16	NA
RVcoll11I830	*Melitaea celadussa*	France	44.559	5.36	WMB1834-13	NA
OXBTGS1286	*Melitaea celadussa*	France	44.7995	5.25968	OXB1568-16	NA
OXBTGS1290	*Melitaea celadussa*	France	44.7995	5.25968	OXB1572-16	NA
OXBTGS1306	*Melitaea celadussa*	France	44.7995	5.25968	OXB1588-16	NA
RVcoll11I949	*Melitaea celadussa*	France	44.81	5.585	WMB1850-13	NA
RVcoll07E394	*Melitaea athalia*	Romania	44.812	25.397	EZROM406-08	HQ004756
RVcoll07D976	*Melitaea athalia*	Romania	44.914	25.641	EZROM405-08	HQ004760
RVcoll15A948	*Melitaea celadussa*	Italy	44.931	10.366	OXB998-15	NA
RVcoll08M222	*Melitaea athalia*	Romania	44.971	25.687	EZRMN099-08	HQ004748
RVcoll15A953	*Melitaea celadussa*	Italy	44.974	10.414	OXB1003-15	NA
RVcoll15A654	*Melitaea celadussa*	Italy	45.328	9.509	OXB1121-15	NA

RVcoll14I118	*Melitaea celadussa*	Italy	45.492	8.313	BIBSA450-15	NA
RVcoll14I193	*Melitaea celadussa*	Italy	45.58647	8.23158	BIBSA1439-16	NA
GWOTD804-12	*Melitaea athalia*	Croatia	45.613	16.75	GWOTD804-12	KX045246
RVcoll15H149	*Melitaea celadussa*	Italy	45.6332	7.3223	EULEP4256-16	NA
RVcoll07E205	*Melitaea celadussa*	Italy	45.667	7.23	NA	NA
RVcoll14U807	*Melitaea celadussa*	Italy	45.7	10.86	OXB844-15	NA
RVcoll14U808	*Melitaea celadussa*	Italy	45.7	10.86	OXB845-15	NA
RVcoll07D295	*Melitaea athalia*	Romania	45.777	25.115	EZROM404-08	HQ004759
GBLAA1948-15	*Melitaea celadussa*	Italy	45.796	10.091	GBLAA1948-15	NA
LEPAA083-16	*Melitaea celadussa*	Switzerland	45.9068	8.92058	NA	MK186560
LEPPA1215-17	*Melitaea celadussa*	Switzerland	46.0061	7.76137	NA	MK186565
RVcoll13U151	*Melitaea celadussa*	Italy	46.02	12.255	OXB725-15	NA
RVcoll13U124	*Melitaea celadussa*	Italy	46.024	12.28	OXB721-15	NA
LEPAA663-16	*Melitaea celadussa*	Switzerland	46.0956	6.98608	NA	MK186570
RVcoll15M133	*Melitaea celadussa*	France	46.116	5.628	OXB1273-15	NA
RVcoll15M140	*Melitaea celadussa*	France	46.116	5.628	OXB1280-15	NA
RVcoll15M147	*Melitaea celadussa*	France	46.116	5.628	OXB1421-15	NA
RVcoll14N054	*Melitaea celadussa*	Italy	46.153	8.333	OXB340-15	NA
LEPAA303-16	*Melitaea celadussa*	Switzerland	46.1783	8.79844	NA	MK186567
LEPAA302-16	*Melitaea celadussa*	Switzerland	46.1798	8.66895	NA	MK186569
RVcoll14K068	*Melitaea celadussa*	Switzerland	46.181	7.417	EULEP2157-15	NA
RVcoll15J251	*Melitaea celadussa*	Switzerland	46.188	8.09289	EULEP4248-16	NA
RVcoll14L242	*Melitaea celadussa*	Italy	46.203	10.808	WMB5111-14	NA
RVcoll15G573	*Melitaea celadussa*	Switzerland	46.20536	8.03352	EULEP4255-16	NA
RVcoll14O001	*Melitaea celadussa*	Italy	46.247	10.834	OXB612-15	NA
RVcoll14L240	*Melitaea celadussa*	Italy	46.263	10.836	WMB5109-14	NA
RVcoll14I580	*Melitaea celadussa*	Italy	46.276	9.885	WMB5033-14	NA

RVcoll14N073	*Melitaea celadussa*	Italy	46.276	9.885	OXB359-15	NA
LEPAA634-16	*Melitaea celadussa*	Switzerland	46.2764	7.37377	NA	MK186568
LEATC245-13	*Melitaea celadussa*	Italy	46.288	11.201	LEATC245-13	NA
RVcoll15G464	*Melitaea celadussa*	Switzerland	46.29313	8.02021	EULEP4260-16	NA
RVcoll15G841	*Melitaea celadussa*	Italy	46.2936	8.27976	EULEP4235-16	NA
RVcoll15G804	*Melitaea celadussa*	Italy	46.2958	8.30077	EULEP4234-16	NA
RVcoll15G530	*Melitaea celadussa*	Switzerland	46.29767	8.06368	EULEP4254-16	NA
RVcoll15G608	*Melitaea celadussa*	Switzerland	46.29767	8.06368	EULEP4233-16	NA
RVcoll14N063	*Melitaea celadussa*	Italy	46.301	9.842	OXB349-15	NA
GWOSZ215-11	*Melitaea celadussa*	Italy	46.3013	11.4457	GWOSZ215-11	KX040384
RVcoll11J148	*Melitaea celadussa*	Switzerland	46.327	7.971	EULEP647-15	NA
RVcoll15H332	*Melitaea celadussa*	Switzerland	46.3339	7.9393	EULEP4238-16	NA
LEPPA1211-17	*Melitaea celadussa*	Switzerland	46.3461	9.58222	NA	MK186561
RVcoll14V894	*Melitaea celadussa*	France	46.36541	5.898327	EULEP4291-16	NA
RVcoll15G134	*Melitaea celadussa*	France	46.36541	5.898327	EULEP4258-16	NA
RVcoll15G136	*Melitaea celadussa*	France	46.36541	5.898327	EULEP4259-16	NA
RVcoll15G145	*Melitaea celadussa*	France	46.36541	5.898327	EULEP4289-16	NA
RVcoll15G160	*Melitaea celadussa*	France	46.36541	5.898327	EULEP4292-16	NA
RVcoll15G184	*Melitaea celadussa*	France	46.36541	5.898327	NA	NA
RVcoll15G187	*Melitaea celadussa*	France	46.36541	5.898327	EULEP4290-16	NA
RVcoll14V302	*Melitaea celadussa*	Italy	46.37312	9.360379	NA	NA
RVcoll15I087	*Melitaea athalia*	Italy	46.3959	13.4342	EULEP4242-16	NA
RVcoll15I041	*Melitaea athalia*	Italy	46.4147	13.4403	EULEP4241-16	NA
LEPPA1212-17	*Melitaea celadussa*	Switzerland	46.4496	6.2022	NA	MK186562
RVcoll15G991	*Melitaea celadussa*	Switzerland	46.4588	8.6818	EULEP4236-16	NA
RVcoll15L848	*Melitaea athalia*	Italy	46.46	13.67	BIBSA1115-15	NA
RVcoll15L850	*Melitaea athalia*	Italy	46.46	13.67	NA	NA

RVcoll15L852	*Melitaea athalia*	Italy	46.46	13.67	BIBSA1116-15	NA
RVcoll13U295	*Melitaea athalia*	Italy	46.482	12.315	NA	NA
RVcoll13U296	*Melitaea athalia*	Italy	46.482	12.315	OXB740-15	NA
RVcoll15H589	*Melitaea celadussa*	Switzerland	46.4969	9.9086	EULEP4239-16	NA
LEATD293-13	*Melitaea celadussa*	Italy	46.52	10.48	LEATD293-13	NA
LEPAA540-16	*Melitaea celadussa*	Switzerland	46.544	6.72157	NA	MK186564
RVcoll11J063	*Melitaea celadussa*	Switzerland	46.566	8.364	EULEP635-15	NA
RVcoll11J064	*Melitaea celadussa*	Switzerland	46.566	8.364	NA	NA
RVcoll13U438	*Melitaea athalia*	Italy	46.589	12.853	OXB747-15	NA
RVcoll13U440	*Melitaea athalia*	Italy	46.589	12.853	NA	NA
PHLAH288-12	*Melitaea celadussa*	Switzerland	46.593	8.656	PHLAH288-12	NA
PHLAC444-10	*Melitaea celadussa*	Italy	46.597	11.439	PHLAC444-10	JF860020
RVcoll15H191	*Melitaea celadussa*	Switzerland	46.65392	8.04986	EULEP4257-16	NA
RVcoll15H751	*Melitaea celadussa*	Switzerland	46.6616	9.5994	EULEP4240-16	NA
RVcoll06M839	*Melitaea athalia*	Romania	46.671	23.567	EZROM403-08	HQ004758
LEASS514-17	*Melitaea athalia*	Austria	46.6833	13.9167	LEASS514-17	NA
RVcoll08M346	*Melitaea athalia*	Romania	46.743	25.664	EZRMN100-08	HQ004749
RVcoll15I495	*Melitaea celadussa*	Austria	46.78895	12.87467	EULEP4244-16	NA
RVcoll08M597	*Melitaea athalia*	Romania	46.793	25.683	EZRMN101-08	HQ004750
RVcoll06K602	*Melitaea athalia*	Romania	46.799	23.959	EZROM402-08	HQ004757
PHLAB360-10	*Melitaea celadussa*	Switzerland	46.833	9.633	PHLAB360-10	HQ968519-SUPPRESSED
RVcoll15I360	*Melitaea athalia*	Austria	46.86858	13.42624	EULEP4243-16	NA
LEPPA1216-17	*Melitaea celadussa*	Switzerland	46.888	9.47257	NA	MK186566
LEASS497-17	*Melitaea athalia*	Austria	46.9508	12.9669	LEASS497-17	NA
PHLAI519-13	*Melitaea celadussa*	Austria	46.963	10.592	PHLAI519-13	NA
LEPPA1213-17	*Melitaea celadussa*	Switzerland	46.9802	9.34459	NA	MK186563
LEPAA436-16	*Melitaea athalia*	Switzerland	46.9938	8.16171	NA	MK186544

LEASS880-17	*Melitaea athalia*	Austria	47.0512	12.8078	LEASS880-17	NA
LEATJ1299-16	*Melitaea athalia*	Austria	47.122	15.488	LEATJ1299-16	NA
RVcoll15I809	*Melitaea athalia*	Austria	47.14822	13.38144	EULEP4247-16	NA
LEATF462-14	*Melitaea athalia*	Austria	47.19	11.1	LEATF462-14	NA
KM572443.1	*Melitaea celadussa*	Austria	47.243	9.872	NA	KM572443.1
PHLSA657-11	*Melitaea celadussa*	Austria	47.243	9.872	PHLSA657-11	KM572443
PHLAB326-10	*Melitaea celadussa*	Austria	47.252	9.686	PHLAB326-10	HQ968488-SUPPRESSED
LEPAA408-16	*Melitaea athalia*	Switzerland	47.3554	7.76953	NA	MK186545
LEASS489-17	*Melitaea athalia*	Austria	47.4278	13.6178	LEASS489-17	NA
RVcoll15I661	*Melitaea athalia*	Austria	47.4597	13.6181	EULEP4245-16	NA
GWOSN694-11	*Melitaea athalia*	Germany	47.557	11.433	GWOSN694-11	KX046864
GWORR680-10	*Melitaea athalia*	Germany	47.5572	11.4333	GWORR680-10	KX040677
LEATJ1296-16	*Melitaea athalia*	Austria	47.56	14.58	LEATJ1296-16	NA
PHLAH287-12	*Melitaea celadussa*	Austria	47.58	9.759	PHLAH287-12	KP253620
RVcoll15I725	*Melitaea athalia*	Austria	47.59278	13.58146	EULEP4246-16	NA
LEATJ1298-16	*Melitaea athalia*	Austria	47.595	13.883	LEATJ1298-16	NA
LEATJ1297-16	*Melitaea athalia*	Austria	47.608	14.746	LEATJ1297-16	NA
RVcoll07C312	*Melitaea athalia*	Romania	47.667	26.65	EZROM693-08	HQ004754
RVcoll07C314	*Melitaea athalia*	Romania	47.667	26.65	EZROM694-08	HQ004755
RVcoll08M103	*Melitaea athalia*	Romania	47.667	26.65	EZRMN372-08	HQ004751
RVcoll08M104	*Melitaea athalia*	Romania	47.667	26.65	EZRMN373-08	HQ004752
RVcoll08M105	*Melitaea athalia*	Romania	47.667	26.65	EZRMN374-08	HQ004753
GWORA2499-09	*Melitaea celadussa*	Germany	47.67	12.04	GWORA2499-09	KX040937
GWOSK889-11	*Melitaea athalia*	Germany	47.7272	11.5451	GWOSK889-11	JN274550-SUPPRESSED
ABOLD023-16	*Melitaea athalia*	Austria	47.75	16.167	ABOLD023-16	NA
GWORR679-10	*Melitaea athalia*	Germany	47.801	11.464	GWORR679-10	KX040599
GWOSN693-11	*Melitaea athalia*	Germany	47.803	11.4622	GWOSN693-11	KX047245

RVcoll16J000	*Melitaea athalia*	Slovakia	48.6139	20.4172	EULEP5036-16	NA
GWORA2497-09	*Melitaea athalia*	Germany	48.878	12.451	GWORA2497-09	HM393215-SUPPRESSED
FBLMU112-09	*Melitaea athalia*	Germany	48.884	13.153	FBLMU112-09	GU707192-SUPPRESSED
GWORA2498-09	*Melitaea athalia*	Germany	48.946	12.855	GWORA2498-09	KX040596
GWOSA739-10	*Melitaea athalia*	Germany	48.99	13.35	GWOSA739-10	HQ565470-SUPPRESSED
GWOSA740-10	*Melitaea athalia*	Germany	48.99	13.35	GWOSA740-10	HQ565471-SUPPRESSED
FBLMT898-09	*Melitaea athalia*	Germany	49.0314	11.9626	FBLMT898-09	GU655013
RVcoll15G067	*Melitaea athalia*	France	49.07129	7.50562	EULEP4250-16	NA
FBLMU119-09	*Melitaea athalia*	Germany	49.1678	11.9452	FBLMU119-09	GU707188
FBLMZ162-12	*Melitaea athalia*	Germany	49.1678	11.9452	FBLMZ162-12	KX040860
RVcoll14I815	*Melitaea athalia*	Slovakia	49.229	20.317	EULEP2015-15	NA
BCZSMLep50476	*Melitaea athalia*	Germany	49.2654	12.3892	NA	NA
FBLMX265-11	*Melitaea athalia*	Germany	49.2654	12.3892	FBLMX265-11	KP870791
RVcoll14V075	*Melitaea athalia*	Ukraine	49.817	35.75	EULEP2380-15	NA
RVcoll14V084	*Melitaea athalia*	Ukraine	50.117	36.067	EULEP2387-15	NA
RVcoll16H716	*Melitaea athalia*	Belgium	50.1388	4.62144	EULEP4820-16	NA
RVcoll15P033	*Melitaea athalia*	Ukraine	50.31	29.11	EULEP4252-16	NA
RVcoll14N413	*Melitaea athalia*	Ukraine	51.01	32.11	EULEP2234-15	NA
RVcoll16I052	*Melitaea athalia*	Poland	52.819	14.2346	EULEP4939-16	NA
RVcoll16J612	*Melitaea athalia*	Russia	54.3856	22.3677	EULEP4909-16	NA
RVcoll15Q076	*Melitaea athalia*	Russia	54.8176	34.5609	EULEP4267-16	NA
RVcoll15Q078	*Melitaea athalia*	Russia	54.8176	34.5609	EULEP4251-16	NA
EULEP347-14	*Melitaea athalia*	Lithuania	55.18	25.23	EULEP347-14	MM23832
EULEP348-14	*Melitaea athalia*	Lithuania	55.213	26.708	EULEP348-14	MM23833
LOWA290-06	*Melitaea athalia*	Russia	56.133	28.667	LOWA290-06	FJ663813
LOWA291-06	*Melitaea athalia*	Russia	56.133	28.667	LOWA291-06	FJ663812
LON203-08	*Melitaea athalia*	Sweden	56.59	16.42	LON203-08	KX049391

EULEP317-14	*Melitaea athalia*	Latvia	56.73	23.88	EULEP317-14	MM23802
RVcoll10A789	*Melitaea athalia*	Estonia	58.1072	26.9178	EULEP4230-16	NA
RVcoll12Z197	*Melitaea athalia*	Sweden	59.612	18.485	EULEP785-15	NA
LEFIJ549-10	*Melitaea athalia*	Finland	59.945	22.4	LEFIJ549-10	JF853664-SUPPRESSED
LON023-08	*Melitaea athalia*	Norway	60.8867	12.2347	LON023-08	KX048533
LEFID245-10	*Melitaea athalia*	Finland	65.782	24.51	LEFID245-10	HM873044-SUPPRESSED
LEFIE848-10	*Melitaea athalia*	Finland	65.861	24.371	LEFIE848-10	HM874567-SUPPRESSED
RVcoll16H415	*Melitaea athalia*	Sweden	67.87876	18.90235	EULEP4784-16	NA
RVcoll18O960	*Melitaea celadussa*	Italy	44.5919	7.0801	NA	NA
RVcoll18P029	*Melitaea celadussa*	Italy	44.5919	7.0801	NA	NA
RVcoll18P030	*Melitaea celadussa*	Italy	44.5919	7.0801	NA	NA
RVcoll18P031	*Melitaea celadussa*	Italy	44.5919	7.0801	NA	NA
RVcoll18P033	*Melitaea celadussa*	Italy	44.5919	7.0801	NA	NA
RVcoll18P041	*Melitaea celadussa*	Italy	46.3	8.3	NA	NA
RVcoll18P047	*Melitaea celadussa*	Italy	46.39	8.42	NA	NA
RVcoll17F943	*Melitaea athalia*	Albania	42.61648	19.67813	NA	NA
RVcoll17E766	*Melitaea celadussa*	France	45.539	2.756	NA	NA
RVcoll17D269	*Melitaea celadussa*	France	45.38743	4.596897	NA	NA
RVcoll17C887	*Melitaea athalia*	Germany	49.64492	7.151968	NA	NA
RVcoll17C750	*Melitaea celadussa*	France	48.03363	4.441152	NA	NA
RVcoll17C668	*Melitaea athalia*	France	47.28357	3.472961	NA	NA
RVcoll17C613	*Melitaea athalia*	France	46.58978	1.263415	NA	NA
RVcoll15M156	*Melitaea celadussa*	France	46.116	5.628	NA	NA
RVcoll15G197	*Melitaea celadussa*	France	46.36541	5.898327	NA	NA
RVcoll13U297	*Melitaea athalia*	Italy	46.482	12.315	BIBSA2099-20	NA
RVcoll14W716	*Melitaea celadussa*	Italy	46.263	10.836	BIBSA2112-20	NA
RVcoll15L869	*Melitaea athalia*	Italy	46.197	13.534	BIBSA2114-20	NA

RVcoll16G235	*Melitaea celadussa*	Italy	46.95	11.854	BIBSA2115-20	NA
RVcoll19E019	*Melitaea celadussa*	Italy	43.303	12.053	BIBSA2151-20	NA
RVcoll19E025	*Melitaea celadussa*	Italy	43.303	12.053	BIBSA2157-20	NA
RVcoll12R008	*Melitaea celadussa*	Italy	37.967	14.75	BIBSA2222-20	NA
RVcoll12R012	*Melitaea celadussa*	Italy	37.97	14.77	BIBSA2223-20	NA
MBMPA005-07	*Melitaea athalia*	Russia	50.485	45.678	MBMPA005-07	NA
MBMPA009-07	*Melitaea athalia*	Russia	50.485	45.678	MBMPA009-07	NA
MBMPA013-07	*Melitaea athalia*	Russia	50.485	45.678	MBMPA013-07	NA
MBMPA020-07	*Melitaea athalia*	Russia	50.485	45.678	MBMPA020-07	NA
MBMPA021-07	*Melitaea athalia*	Russia	50.485	45.678	MBMPA021-07	NA
MBMPA036-07	*Melitaea athalia*	Russia	50.485	45.678	MBMPA036-07	NA
MBMPA103-07	*Melitaea athalia*	Russia	54.8	37.4	MBMPA103-07	NA
MBMPA107-07	*Melitaea athalia*	Russia	54.8	37.4	MBMPA107-07	NA
MBMPA112-07	*Melitaea athalia*	Russia	54.8	37.4	MBMPA112-07	NA
MBMPA118-07	*Melitaea athalia*	Russia	54.8	37.4	MBMPA118-07	NA
MBMPA121-07	*Melitaea athalia*	Russia	54.8	37.4	MBMPA121-07	NA
MBMPA127-07	*Melitaea athalia*	Russia	54.8	37.4	MBMPA127-07	NA
MBMPA128-07	*Melitaea athalia*	Russia	54.8	37.4	MBMPA128-07	NA
MBMPA131-07	*Melitaea athalia*	Russia	54.8	37.4	MBMPA131-07	NA
MBMPA139-07	*Melitaea athalia*	Russia	54.8	37.4	MBMPA139-07	NA
MBMPA141-07	*Melitaea athalia*	Russia	54.8	37.4	MBMPA141-07	NA
MBMPA142-07	*Melitaea athalia*	Russia	54.8	37.4	MBMPA142-07	NA
MBMPA144-07	*Melitaea athalia*	Russia	54.8	37.4	MBMPA144-07	NA
MBMPA145-07	*Melitaea athalia*	Russia	54.8	37.4	MBMPA145-07	NA
MBMPA149-07	*Melitaea athalia*	Russia	54.8	37.4	MBMPA149-07	NA
MBMPA152-07	*Melitaea athalia*	Russia	54.8	37.4	MBMPA152-07	NA
MBMPA153-07	*Melitaea athalia*	Russia	54.8	37.4	MBMPA153-07	NA

MBMPA174-07	*Melitaea athalia*	Russia	50.485	45.678	MBMPA174-07	NA
MBMPA176-07	*Melitaea athalia*	Russia	50.485	45.678	MBMPA176-07	NA
MBMPA185-07	*Melitaea athalia*	Russia	50.485	45.678	MBMPA185-07	NA
MBMPA186-07	*Melitaea athalia*	Russia	50.485	45.678	MBMPA186-07	NA
MBMPA200-09	*Melitaea athalia*	Russia	55.42	60.46	MBMPA200-09	NA
MBMPA204-09	*Melitaea athalia*	Russia	55.42	60.46	MBMPA204-09	NA
MBMPA205-09	*Melitaea athalia*	Russia	55.42	60.46	MBMPA205-09	NA
MBMPA206-09	*Melitaea athalia*	Russia	55.42	60.46	MBMPA206-09	NA
MBMPA208-09	*Melitaea athalia*	Russia	55.42	60.46	MBMPA208-09	NA
MBMPA223-09	*Melitaea athalia*	Russia	56.48	59.54	MBMPA223-09	NA
MBMPA225-09	*Melitaea athalia*	Russia	56.372	61.01	MBMPA225-09	NA
MBMPA226-09	*Melitaea athalia*	Russia	56.372	61.01	MBMPA226-09	NA
MBMPA230-09	*Melitaea athalia*	Russia	56.372	61.01	MBMPA230-09	NA
MBMPA232-09	*Melitaea athalia*	Russia	54.4	59.4	MBMPA232-09	NA
MBMPA234-09	*Melitaea athalia*	Russia	54.4	59.4	MBMPA234-09	NA
MBMPA235-09	*Melitaea athalia*	Russia	54.4	59.4	MBMPA235-09	NA
MBMPA237-09	*Melitaea athalia*	Russia	54.4	59.4	MBMPA237-09	NA
MBMPA001-07	*Melitaea athalia*	Russia	50.485	45.678	MBMPA001-07	NA
MBMPA003-07	*Melitaea athalia*	Russia	50.485	45.678	MBMPA003-07	NA
MBMPA006-07	*Melitaea athalia*	Russia	50.485	45.678	MBMPA006-07	NA
MBMPA007-07	*Melitaea athalia*	Russia	50.485	45.678	MBMPA007-07	NA
MBMPA014-07	*Melitaea athalia*	Russia	50.485	45.678	MBMPA014-07	NA
MBMPA015-07	*Melitaea athalia*	Russia	50.485	45.678	MBMPA015-07	NA
MBMPA019-07	*Melitaea athalia*	Russia	50.485	45.678	MBMPA019-07	NA
MBMPA027-07	*Melitaea athalia*	Russia	50.485	45.678	MBMPA027-07	NA
MBMPA034-07	*Melitaea athalia*	Russia	50.485	45.678	MBMPA034-07	NA
MBMPA099-07	*Melitaea athalia*	Russia	54.8	37.4	MBMPA099-07	NA

MBMPA100-07	*Melitaea athalia*	Russia	54.8	37.4	MBMPA100-07	NA
MBMPA102-07	*Melitaea athalia*	Russia	54.8	37.4	MBMPA102-07	NA
MBMPA104-07	*Melitaea athalia*	Russia	54.8	37.4	MBMPA104-07	NA
MBMPA105-07	*Melitaea athalia*	Russia	54.8	37.4	MBMPA105-07	NA
MBMPA109-07	*Melitaea athalia*	Russia	54.8	37.4	MBMPA109-07	NA
MBMPA113-07	*Melitaea athalia*	Russia	54.8	37.4	MBMPA113-07	NA
MBMPA114-07	*Melitaea athalia*	Russia	54.8	37.4	MBMPA114-07	NA
MBMPA116-07	*Melitaea athalia*	Russia	54.8	37.4	MBMPA116-07	NA
MBMPA119-07	*Melitaea athalia*	Russia	54.8	37.4	MBMPA119-07	NA
MBMPA120-07	*Melitaea athalia*	Russia	54.8	37.4	MBMPA120-07	NA
MBMPA122-07	*Melitaea athalia*	Russia	54.8	37.4	MBMPA122-07	NA
MBMPA123-07	*Melitaea athalia*	Russia	54.8	37.4	MBMPA123-07	NA
MBMPA124-07	*Melitaea athalia*	Russia	54.8	37.4	MBMPA124-07	NA
MBMPA125-07	*Melitaea athalia*	Russia	54.8	37.4	MBMPA125-07	NA
MBMPA133-07	*Melitaea athalia*	Russia	54.8	37.4	MBMPA133-07	NA
MBMPA134-07	*Melitaea athalia*	Russia	54.8	37.4	MBMPA134-07	NA
MBMPA135-07	*Melitaea athalia*	Russia	54.8	37.4	MBMPA135-07	NA
MBMPA137-07	*Melitaea athalia*	Russia	54.8	37.4	MBMPA137-07	NA
MBMPA148-07	*Melitaea athalia*	Russia	54.8	37.4	MBMPA148-07	NA
MBMPA150-07	*Melitaea athalia*	Russia	54.8	37.4	MBMPA150-07	NA
MBMPA151-07	*Melitaea athalia*	Russia	54.8	37.4	MBMPA151-07	NA
MBMPA155-07	*Melitaea athalia*	Russia	54.8	37.4	MBMPA155-07	NA
MBMPA156-07	*Melitaea athalia*	Russia	54.8	37.4	MBMPA156-07	NA
MBMPA177-07	*Melitaea athalia*	Russia	50.485	45.678	MBMPA177-07	NA
MBMPA181-07	*Melitaea athalia*	Russia	50.485	45.678	MBMPA181-07	NA
MBMPA188-07	*Melitaea athalia*	Russia	50.485	45.678	MBMPA188-07	NA
MBMPA199-09	*Melitaea athalia*	Russia	55.42	60.46	MBMPA199-09	NA

MBMPA201-09	*Melitaea athalia*	Russia	55.42	60.46	MBMPA201-09	NA
MBMPA202-09	*Melitaea athalia*	Russia	55.42	60.46	MBMPA202-09	NA
MBMPA207-09	*Melitaea athalia*	Russia	55.42	60.46	MBMPA207-09	NA
MBMPA227-09	*Melitaea athalia*	Russia	56.372	61.01	MBMPA227-09	NA
MBMPA228-09	*Melitaea athalia*	Russia	54.4	59.4	MBMPA228-09	NA
MBMPA229-09	*Melitaea athalia*	Russia	54.4	59.4	MBMPA229-09	NA
MBMPA233-09	*Melitaea athalia*	Russia	54.4	59.4	MBMPA233-09	NA
MBMPA238-09	*Melitaea athalia*	Russia	54.4	59.4	MBMPA238-09	NA
EZHBA657-07	*Melitaea athalia*	Russia	54.6	83.3	EZHBA657-07	NA
MBMPA029-07	*Melitaea athalia*	Russia	50.485	45.678	MBMPA029-07	NA
MBMPA032-07	*Melitaea athalia*	Russia	50.485	45.678	MBMPA032-07	NA
MBMPA035-07	*Melitaea athalia*	Russia	50.485	45.678	MBMPA035-07	NA
MBMPA095-07	*Melitaea athalia*	Russia	54.8	37.4	MBMPA095-07	NA
MBMPA096-07	*Melitaea athalia*	Russia	54.8	37.4	MBMPA096-07	NA
MBMPA097-07	*Melitaea athalia*	Russia	54.8	37.4	MBMPA097-07	NA
MBMPA098-07	*Melitaea athalia*	Russia	54.8	37.4	MBMPA098-07	NA
MBMPA101-07	*Melitaea athalia*	Russia	54.8	37.4	MBMPA101-07	NA
MBMPA106-07	*Melitaea athalia*	Russia	54.8	37.4	MBMPA106-07	NA
MBMPA108-07	*Melitaea athalia*	Russia	54.8	37.4	MBMPA108-07	NA
MBMPA110-07	*Melitaea athalia*	Russia	54.8	37.4	MBMPA110-07	NA
MBMPA111-07	*Melitaea athalia*	Russia	54.8	37.4	MBMPA111-07	NA
MBMPA115-07	*Melitaea athalia*	Russia	54.8	37.4	MBMPA115-07	NA
MBMPA117-07	*Melitaea athalia*	Russia	54.8	37.4	MBMPA117-07	NA
MBMPA126-07	*Melitaea athalia*	Russia	54.8	37.4	MBMPA126-07	NA
MBMPA129-07	*Melitaea athalia*	Russia	54.8	37.4	MBMPA129-07	NA
MBMPA130-07	*Melitaea athalia*	Russia	54.8	37.4	MBMPA130-07	NA
MBMPA132-07	*Melitaea athalia*	Russia	54.8	37.4	MBMPA132-07	NA

MBMPA136-07	*Melitaea athalia*	Russia	54.8	37.4	MBMPA136-07	NA
MBMPA138-07	*Melitaea athalia*	Russia	54.8	37.4	MBMPA138-07	NA
MBMPA140-07	*Melitaea athalia*	Russia	54.8	37.4	MBMPA140-07	NA
MBMPA143-07	*Melitaea athalia*	Russia	54.8	37.4	MBMPA143-07	NA
MBMPA146-07	*Melitaea athalia*	Russia	54.8	37.4	MBMPA146-07	NA
MBMPA147-07	*Melitaea athalia*	Russia	54.8	37.4	MBMPA147-07	NA
MBMPA154-07	*Melitaea athalia*	Russia	54.8	37.4	MBMPA154-07	NA
MBMPA157-07	*Melitaea athalia*	Russia	54.8	37.4	MBMPA157-07	NA
MBMPA169-07	*Melitaea athalia*	Russia	50.485	45.678	MBMPA169-07	NA
MBMPA182-07	*Melitaea athalia*	Russia	50.485	45.678	MBMPA182-07	NA
MBMPA183-07	*Melitaea athalia*	Russia	50.485	45.678	MBMPA183-07	NA
MBMPA203-09	*Melitaea athalia*	Russia	55.42	60.46	MBMPA203-09	NA
MBMPA219-09	*Melitaea athalia*	Russia	51.285	57.253	MBMPA219-09	NA
MBMPA220-09	*Melitaea athalia*	Russia	51.285	57.253	MBMPA220-09	NA
MBMPA224-09	*Melitaea athalia*	Russia	56.48	59.54	MBMPA224-09	NA
MBMPA231-09	*Melitaea athalia*	Russia	54.4	59.4	MBMPA231-09	NA

Bibliography

1. Dincă V, Zakharov E V, Hebert PDN, Vila R. Complete DNA barcode reference library for a country's butterfly fauna reveals high performance for temperate Europe. Proc R Soc B Biol Sci. 2011 Feb;278(1704):347–55.

2. Dincă V, Montagud S, Talavera G, Hernández-Roldán J, Munguira ML, García-Barros E, et al. DNA barcode reference library for Iberian butterflies enables a continental-scale preview of potential cryptic diversity. Sci Rep. 2015 Dec;5(1):12395.

3. Dincă V, Dapporto L, Somervuo P, Vodă R, Cuvelier S, Gascoigne-Pees M, et al. High resolution DNA barcode library for European butterflies reveals continental patterns of mitochondrial genetic diversity. Commun Biol. 2021;4(1):1–11.

4. Kumar S, Stecher G, Li M, Knyaz C, Tamura K. MEGA X: molecular evolutionary genetics analysis across computing platforms. Mol Biol Evol. 2018;35(6):1547–9.

5. Van Oorschot H, Coutsis JG. The Genus *Melitaea* Fabricius, 1807: Taxonomy and Systematics with Special Reference to the Male Genitalia:(Lepidoptera, Nymphalidae, Nymphalinae). Tshikolovets Publications; 2014.

6. Dapporto L, Ramazzotti M, Fattorini S, Talavera G, Vila R, Dennis RLH. recluster: an unbiased clustering procedure for beta-diversity turnover. Ecography (Cop). 2013;36(10):1070–5.

7. Robinson GS. The preparation of slides of Lepidoptera genitalia with special reference to the Microlepidoptera. Entomol Gaz. 1976;27(2):127–32.

8. Higgins LG. Classification of European butterflies. Collins; 1975.

9. Chessel D, Dufour A-B, Dray S, Lobry JR, Ollier S, Pavoine S, et al. ade4. 2017. Available from: http://pbil.univ-lyon1.fr/ADE-4/home.php?lang=eng

10. Lee KM, Kivelä SM, Ivanov V, Hausmann A, Kaila L, Wahlberg N, et al. Information dropout patterns in restriction site associated DNA phylogenomics and a comparison with multilocus Sanger data in a species-rich moth genus. Syst Biol. 2018;67(6):925–39.
